# Prognostic factors for progression of osteoarthritis of the hip: a systematic review

**DOI:** 10.1186/s13075-019-1969-9

**Published:** 2019-08-23

**Authors:** C. H. Teirlinck, D. M. J. Dorleijn, P. K. Bos, J. B. M. Rijkels-Otters, S. M. A. Bierma-Zeinstra, P. A. J. Luijsterburg

**Affiliations:** 1 Department of General Practice, Erasmus MC University Medical Center, P.O. Box 2040, 3000 CA Rotterdam, the Netherlands; 2 Department of Orthopedics, Erasmus MC University Medical Center, P.O. Box 2040, 3000 CA Rotterdam, the Netherlands

**Keywords:** Osteoarthritis, Hip, Prognostic factors, Progression, Systematic review

## Abstract

**Background:**

Predicting which patients with hip osteoarthritis are more likely to show disease progression is important for healthcare professionals. Therefore, the aim of this review was to assess which factors are predictive of progression in patients with hip osteoarthritis.

**Methods:**

A literature search was made up until 14 March 2019. Included were cohort and case-control studies evaluating the association between factors and progression (either clinical, radiological, or THR). Excluded were studies with a follow-up < 1 year or specific underlying pathologies of osteoarthritis. Risk of bias was assessed using the QUIPS tool. A best-evidence synthesis was conducted.

**Results:**

We included 57 articles describing 154 different factors. Of these, a best-evidence synthesis was possible for 103 factors, separately for clinical and radiological progression, and progression to total hip replacement. We found strong evidence for more clinical progression in patients with comorbidity and more progression to total hip replacement for a higher Kellgren and Lawrence grade, superior or (supero) lateral femoral head migration, and subchondral sclerosis. Strong evidence for no association was found regarding clinical progression for gender, social support, pain medication, quality of life, and limited range of motion of internal rotation or external rotation. Also, strong evidence for no association was found regarding radiological progression for the markers CTX-I, COMP, NTX-I, PINP, and PIIINP and regarding progression to total hip replacement for body mass index.

**Conclusion:**

Strong evidence suggested that 4 factors were predictive of progression of hip osteoarthritis, whereas 12 factors were not predictive of progression. Evidence for most of the reported factors was either limited or conflicting.

**Protocol registration:**

PROSPERO, CRD42015010757

**Electronic supplementary material:**

The online version of this article (10.1186/s13075-019-1969-9) contains supplementary material, which is available to authorized users.

## Background

The hip is the third joint most commonly affected by osteoarthritis (OA) [1]. No therapeutic cure exists for hip OA. Therefore, predicting which patients with hip OA are more likely to progress in their disease is of special interest, particularly if these predictive factors are potentially modifiable.

In 2002, Lievense et al. published a systematic review in which they identified several predictive factors for the progression of hip OA [2]. They used a best-evidence synthesis to draw conclusions about the available evidence per factor. Strong evidence was found for more rapid progression in patients with a superior or superolateral migration of the femoral head or an atrophic bone response. Conversely, strong evidence was found for no association between progression of hip OA and obesity. In 2009, Wright et al. also reviewed the known prognostic factors and their quality of evidence [3]. They concluded that only a few factors are strongly associated with the progression of hip OA, i.e., age, joint space width, migration of the femoral head, femoral osteophytes, bony sclerosis, Kellgren and Lawrence (K-L) grade 3, hip pain at baseline, and a Lequesne index score > 10. In that review, acetabular osteophytes showed no association with progression. Furthermore, de Rooij et al. studied the factors predicting the course of pain and function. They found strong evidence that higher comorbidity count and lower vitality predict a worsening of physical function [4]. Although all reviews described additional predictive factors, the evidence for these factors was either limited or conflicting.

Since the literature search of Wright et al. (in October 2008) and de Rooij et al. (in July 2015) more research on prognostic factors of hip OA have been conducted, and new methods to assess and review prognostic studies have been developed [5].

Therefore, the aim of this present study was to systematically review the evidence of patient, health, and diagnostic variables associated with the progression of hip OA.

## Methods

### Search of the literature

A search was made in the databases of Embase, MEDLINE (OvidSP), Web-of-Science, Cochrane Library, PubMed publisher, and Google Scholar from the inception of the database until 14 March 2019, using the keywords *hip*, *osteoarthritis*, and *prognosis* (and their synonyms). We excluded congress abstracts and editorial letters from our search by setting these as limits to restrain the number of found citations without losing valuable citations. The reference lists of relevant articles were screened for additional relevant studies. A complete syntax of the search can be found in Additional file 1. The process of the search was assisted and partly conducted by an experienced medical librarian.

### Criteria for selection of studies

The following are the criteria for the selection of studies:

1. The study should investigate the factors associated with the progression of hip OA.
2. The article was written in English, Dutch, German, French, Spanish, Italian, Danish, Norwegian, or Swedish. These languages were sufficiently mastered by at least two reviewers.
3. The article was available in full text.
4. Patients in the study reported complaints like pain, disability, or stiffness of the hip, suspected or confirmed (radiographic or clinical criteria) to originate from OA of the hip.
5. The study design was a cohort or a case-control study or a randomized controlled trial in which the estimation of the prognostic factor was adjusted for the intervention or only investigated in the control group.
6. Progression was determined radiographically or clinically. Radiographic progression could be determined by, for example, X-ray or MRI. Examples of clinical progression were worsening of pain or function or reaching the point of indication for total hip replacement (THR).
7. Follow-up should be at least 1 year (based on the recommendations for measuring structural progression [6]).
8. The study was excluded if the population under investigation had a specific underlying pathology, such as trauma (fractures), infection, rheumatoid arthritis, ankylosing spondylitis, Perthes’ disease, tuberculosis, hemochromatosis, sickle cell disease, Cushing’s syndrome, and femoral head necrosis.

### Selection of studies

CHT screened all the titles and abstracts and excluded articles that did not investigate patients with OA of the hip. Secondly, CHT and PAJL independently selected the titles and abstracts using the selection criteria to decide which articles required the retrieval of full text; in case of disagreement, the full text was retrieved. Then, all full texts were independently assessed by CHT and PAJL to include all relevant studies according to the selection criteria. In case of disagreement and both reviewers were unable to reach consensus, SMABZ made the final decision.

### Data extraction

Information on the design, setting, study population (e.g., recruitment period, age, gender, definition of hip OA), number of participants, follow-up period, loss to follow-up, prognostic factors, assessment of progression, outcomes, and strength of association were extracted using standardized forms by CHT and checked by PAJL.

Prognostic factors were divided into patient variables, disease characteristics, and chemical or imaging markers. Outcomes were divided into clinical progression, radiographic progression, or (indication for) receiving a THR.

If outcomes were measured at several follow-up moments, all moments were extracted. After the collection of all data, the follow-up moments that were in the closest range to each other were used for the evidence synthesis.

### Risk of bias assessment

The quality of all included cohort studies was evaluated using the QUIPS tool [5, 7]. Studies were assessed on six domains: study participation, study attrition, prognostic factor measurement, outcome measurement, study confounding, and statistical analysis and reporting. An overview of all domains and their items is presented in Additional file 2. Each study was independently scored by CHT and by a second reviewer (DMJD, SMABZ, PKB, JBMRO, or PAJL). In case of disagreement, they attempted to reach consensus; if this failed, a third reviewer (JBMRO or PAJL) made the final decision.

### Evidence synthesis

A meta-analysis was considered if clinical heterogeneity was low, with respect to the study population, the risk of bias, and the definition of prognostic factors and defined hip OA progression. In case of a meta-analysis, an adjusted GRADE assessment for prognostic research was used to determine the strength of the evidence [8].

If the level of heterogeneity of the studies was high, we refrained from pooling in the main analysis and performed a qualitative evidence synthesis. Associations were categorized as positive, negative, or no association. Ranking of the levels of evidence was based on Lievense et al. [2] and Davis et al. [9]:

1. Strong evidence: consistent findings (≥ 75% of the studies showing the same direction of the association) in two or more studies with a low risk of bias in all domains of the QUIPS tool
2. Moderate evidence: consistent findings in more than two studies with a moderate or high risk of bias in one or more domains of the QUIPS tool or consistent findings in two studies, of which one study has a low risk of bias in all domains of the QUIPS tool
3. Limited evidence: one study with a low risk of bias in all domains of the QUIPS tool or two studies with a moderate or high risk of bias in one or more domains of the QUIPS tool
4. Conflicting evidence: < 75% of the studies showing the same direction of the association

If a prognostic factor was described in two different articles that investigated the same study cohort and outcome of progression, one study was selected to include in the evidence synthesis. In this case, we selected the article according to a decision tree: (1) lowest risk of bias, (2) prognostic factor is the primary outcome of the study, and (3) the largest number of participants.

### Post hoc changes to the study protocol

After contact with one of the developers of the QUIPS tool, we learned that it is not validated to judge the risk of bias of case-control studies and would probably not adequately take into account the higher risk of recall bias and the selection bias of case-control studies. Therefore, we decided to exclude case-control studies from our evidence synthesis, except for nested case-control studies. Nested case-control studies are less prone to selection and recall bias because of the underlying known cohort [10], which can be judged using the QUIPS tool.

## Results

### Included studies

**Fig. 1:**
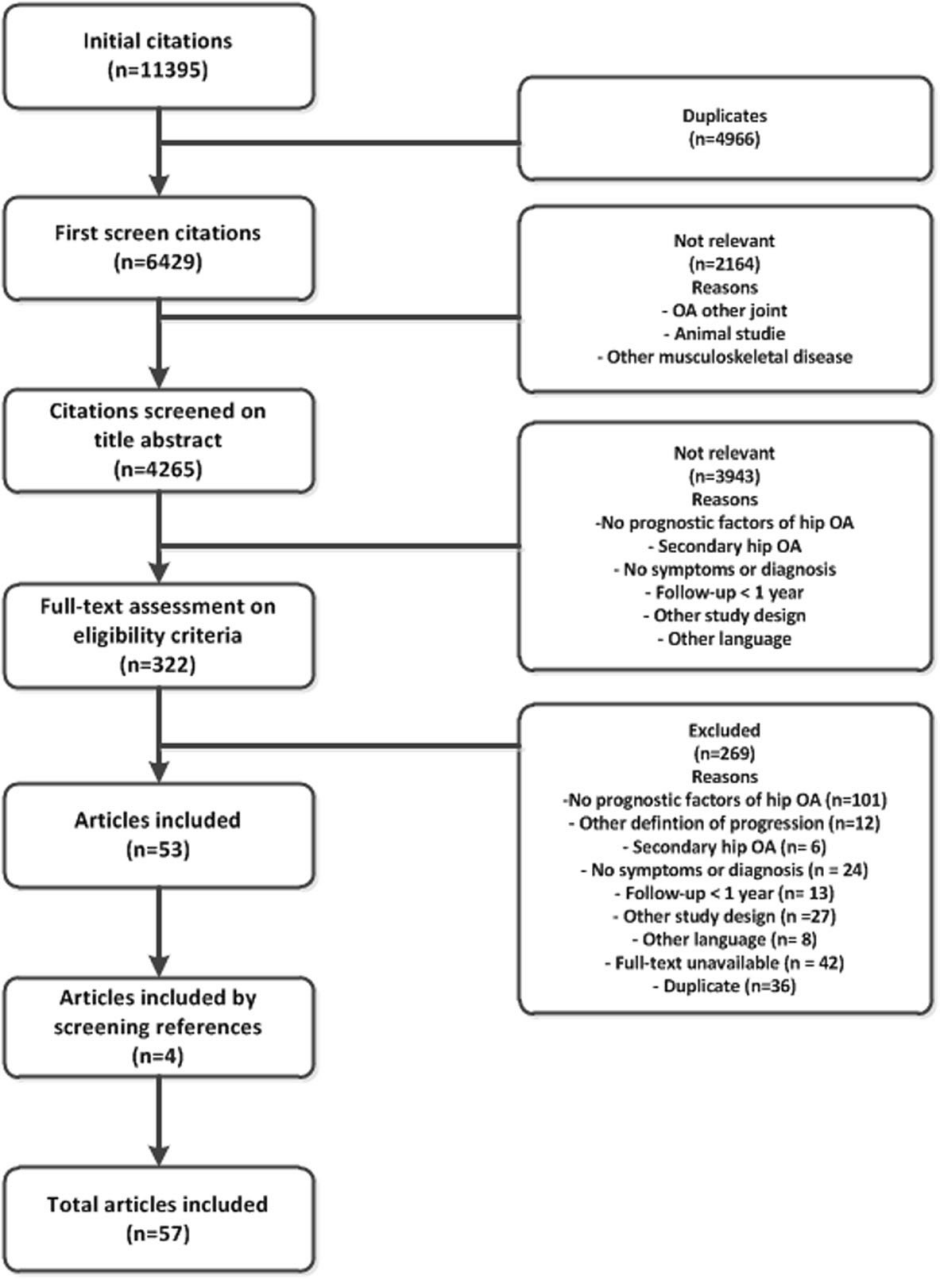
Flowchart of the search and selection of studies

The initial search yielded 6429 citations of which 57 articles were finally included. Figure 1 shows the reasons for the study exclusion, and Table 1 presents a brief overview of the characteristics of the 57 included studies (a more extensive overview is available in Additional file 3). Of the 57 studies, 48 were cohort studies (37 with a prospective design), 4 were nested case-control studies, and 5 were case-control studies. These last 5 studies were excluded from the evidence synthesis for the reasons mentioned above.

**Table 1:** Characteristics of the selected studies

Study	Design	Participants in the cohort (*n*)	Assessment of progression	Follow-up period
Agricola et al. [11]	Prospective cohort (CHECK)	1002 (analyzed 723 patients)	THR	5 years
Agricola et al. [12]	Prospective cohort (CHECK)	1002 (analyzed 550 women)	THR due to OA	5 years
Agricola et al. [12]	Nested case-control (Chingford cohort)	1003 (analyzed 114)	THR due to OA	19 years
Auquier et al. [13]	Retrospective cohort	131	Increase in stage of pain and function, stages minimal, moderate, moderate-severe, severe	6–23 years
Barr et al. [14]	Case-control	195 (analyzed 102 patients)	THR (compared to non-progression hips: increase of ≤ 1 K-L grade)	5 years
Bastick et al. [15]	Prospective cohort (CHECK)	545 (analyzed 363 patients)	NRS score for pain, group moderate progression compared to mild pain. Groups based on LCGA	5 years
Bastick et al. [16]	Prospective cohort (CHECK)	588 (analyzed 538)	THR	5 years
Bergink et al. [17]	Prospective cohort (Rotterdam I)	176	1. Increase ≥ 1 K-L grade 2. Decrease ≥ 1 mm of joint space	Average 8.4 years
Birn et al. [18]	Case-control	94 (5 cases, 89 controls)	Rapidly destructive OA: > 2 mm or > 50% JSN/year	NR
Birrell et al. [19]	Prospective cohort	195	Time to being put on a waiting list for THR	36 months
Bouyer et al. [20]	Prospective cohort (KHOALA)	242 (analyzed 133 patients)	1. Increase ≥ 1 K-L grade 2. Increase ≥ 1 JSN score 3. Time to THR	3 years
Castano Betancourt et al. [21]	Prospective cohort (GOAL)	189	JSN ≥ 20% compared to baseline or THR	2 years
Chaganti et al. [22]	Nested case-control (SOF)	168 cases and 173 controls	Decrease in MJS of 0.5 mm, increase of ≥ 1 in summary grade, increase ≥ 2 in total osteophyte score, or THR for OA	Average 8.3 years
Chevalier et al. [23]	Prospective cohort	30	Rapid evolution: JSN > 0.6 mm/year	1 year
Conrozier et al. [24]	Case-control	104 (analyzed 10 cases, 23 controls)	Rapidly progressive hip OA: severe hip pain, symptom onset within the last 2 years, annual rate of JSN > 1 mm, ESR < 20 mm/h, absence of detectable inflammatory or crystal-induced joint disease	NR
Conrozier et al. [25]	Retrospective cohort	89	Radiographic: YMN, calculated from MJS in mm/year	18–300 months
Conrozier et al. [26]	Prospective cohort	48	JSN in mm/year	1 year
Danielsson [27, 28]	Prospective cohort	168	1. Increase in pain index 0–5 2. Operation because of hip OA 3. Increase in radiographic index 0–10	8–12 years
van Dijk et al. [29]	Prospective cohort	123	1. Decrease in WOMAC function 2. Increase in seconds of timed walking test	3 years
van Dijk et al. [30]	Prospective cohort	123	1. Decrease in WOMAC function 2. Increase in seconds of timed walking test	3 years
Dorleijn et al. [31]	Prospective cohort (GOAL)	222 (analyzed 111 patients)	VAS score for pain, group highly progressive compared to mild pain groups based on LCGA	2 years
Dougados et al. [32]	Prospective cohort (ECHODIAH)	508 (analyzed 461 patients)	Radiological: ≥ 0.6 mm decrease in JSW	1 year
Dougados et al. [33]	Prospective cohort (ECHODIAH)	508 (analyzed 463 patients)	Radiological: > 0.5 mm decrease in JSW	2 years
Dougados et al. [34]	Prospective cohort	508	Time to the requirement of THR	3 years
Fukushima et al. [35]	Prospective cohort	20	Increase in Tönnis grade	25 months
Golightly et al. [36]	Prospective cohort (Johnston County)	1453	Increase in K-L grade or increase in hip symptoms (mild, moderate, severe)	3–13 years
Gossec et al. [37]	Prospective cohort	741 (analyzed 505 patients)	THR	2 years
Hartofilakidis et al. [38]	Retrospective cohort	210	THR	2 to > 10 years
Hawker et al. [39]	Prospective cohort	2128	Time to THR	6.1 years
Hoeven et al. [40]	Prospective cohort (Rotterdam I)	5650 (number analyzed: NR)	Increase ≥ 1 K-L grade baseline to follow-up	10 years
Holla et al. [41]	Prospective cohort (CHECK)	588	Moving into a higher group (quintiles of WOMAC-PF 0–68) or remaining within the three highest groups	2 years
Juhakoski et al. [42]	Prospective cohort	118	1. WOMAC pain (0–100) 2. WOMAC function (0–100)	2 years
Kalyoncu et al. [43]	Retrolective cohort (ECHODIAH)	192	THR	10 years
Kelman et al. [44]	Nested case-control (SOF)	396 (cases 197, controls 199)	Decrease in minimum joint space of ≥ 0.5 mm, an increase of ≥ 1 in the summary grade, an increase of ≥ 2 in total osteophyte score, or THR	8.3 years
Kerkhof et al. [45]	Prospective cohort (Rotterdam I)	1610	Radiologic: JSN ≤ 1.0 mm or THR during follow-up	NR
Kopec et al. [46]	Prospective cohort (Johnston County)	1590 (analyzed 571 people)	Increase ≥ 1 in K-L grade	3–13 years
Lane et al. [47]	Prospective cohort (SOF)	745	Decrease in minimum joint space of ≥ 0.5 mm, an increase of ≥ 1 in the summary grade, an increase of ≥ 2 in total osteophyte score, or THR	8 years
Lane et al. [48]	Nested case-control (SOF)	342	Radiological: decrease in minimum joint space of ≥ 0.5 mm, an increase of ≥ 1 in the summary grade, an increase of ≥ 2 in total osteophyte score, or THR	8.3 years
Laslett et al. [49]	Prospective cohort (TasOAC)	1099 (analyzed 765 people)	WOMAC pain (0–100)	2–4 years
Ledingham 1993 [50]	Prospective cohort	136	1. Global assessment of radiographic change 2. THR	3–73 months
Lievense et al. [51]	Prospective cohort	224 (analyzed 163 patients)	THR	5.8 years
Maillefert et al. [52]	Prospective cohort (ECHODIAH)	508	1. Decrease in JSW > 50% during the first year follow-up 2. THR in 1–5 years of follow-up	5 years
Mazieres et al. [53]	Prospective cohort (ECHODIAH)	507 (analyzed 333 patients)	JSN ≥ 0.5 mm or THP	3 years
Nelson et al. [54]	Prospective cohort (Johnston County)	309	1. Increase in K-L grade 2. Increase in osteophyte severity grade 3. Increase in JSN severity grade	5 years
Perry et al. [55]	Case-control	44	Radiographic: progressive deterioration	5–14 years
Peters et al. [56]	Prospective cohort	587 (analyzed 214 patients)	New Zealand score 0–80 (combination of pain and function)	7 years
Pisters et al. [57]	Prospective cohort	149	Increase in WOMAC function on average over time (measured at 1, 2, 3, 5 years)	5 years
Pollard 201et al. 2 [58]	Prospective cohort	264	Signs on examination of hip OA or symptoms at baseline and signs and symptoms at follow-up	5 years
Reijman et al. [59]	Prospective cohort (Rotterdam I)	1235	JSN ≥ 1.0 mm in at least 1 of 3 compartments (lateral, superior, axial)	6.6 years
Reijman et al. [60]	Prospective cohort (Rotterdam I)	1904	Radiologic: JSN ≤ 1.0 mm or THR during follow-up	6.6 years
Reijman et al. [61]	Prospective cohort (Rotterdam I)	1676	1. JSN of ≥ 1 mm 2. JSN of ≥ 1.5 mm 3. Increase of ≥ 1 K-L grade	6.6 years
Solignac [62]	Prospective cohort (ECHODIAH)	507 (analyzed 333 patients)	JSN ≥ 0.5 mm or THP	3 years
van Spil et al. [63]	Prospective cohort (CHECK)	1002 (analyzed 178 patients)	Radiographic: ≥ 1 K-L grade increase	5 years
Thompson et al. [64]	Case-control	34 cases, controls: NR	Rapidly progressive OA: loss of bone or a combined loss of bone and articular cartilage at rate > 5 mm per year	18 months
Tron et al. [65]	Retrospective cohort	39	Mean annual JSN in mm	NR
Verkleij et al. [66]	Prospective cohort (GOAL)	222 (analyzed 111 patients)	VAS score for pain, group highly progressive compared to mild pain, groups based on LCGA	2 years
Vinciguerra et al. [67]	Retrospective cohort	149	Time to THR	Variable

*NR* not reported, *OA* osteoarthritis *THR* total hip replacement, *K-L grade* Kellgren and Lawrence grade, *MJS* minimum joint space, *JSN* joint space narrowing, *JSW* joint space width, *YMN* yearly mean narrowing, *LCGA* latent class growth analysis, *ESR* erythrocyte sedimentation rate, *NRS* numeric rating scale, *VAS* visual analog scale

### Risk of bias assessment

In 68% of all assessed domains from all studies, there was an immediate consensus between the reviewers (Cohen’s kappa 0.375, linear weighted kappa 0.484). In 9 assessments of a domain (3%) in 6 different studies, a third reviewer made the final judgment. In total, 15 studies scored a low risk of bias in all domains [15, 16, 21, 29, 30, 32, 34, 37, 41, 44, 47, 49, 53, 57, 63] (Table 2).

**Table 2:** Risk of bias assessment summary (QUIPS)

Study	Study participation	Study attrition	Prognostic factor measurement	Outcome measurement	Study confounding	Statistical analysis and reporting
Agricola et al. [11]	Low	Low	Moderate	Low	Low	Low
Agricola et al. [12]	Low	Low	Moderate	Low	Moderate	Low
Auquier et al. [13]	Moderate	Moderate	Low	Moderate	High	Moderate
Bastick et al. [15]	*Low*	*Low*	*Low*	*Low*	*Low*	*Low*
Bastick et al. [16]	*Low*	*Low*	*Low*	*Low*	*Low*	*Low*
Bergink et al. [17]	Low	Moderate	Moderate	Low	Low	Moderate
Bouyer et al. [20]	Low	High	Moderate	Moderate	Low	Low
Birrell et al. [19]	Low	Low	Moderate	Low	Low	Low
Castano Betancourt et al. [21]	*Low*	*Low*	*Low*	*Low*	*Low*	*Low*
Chaganti et al. [22]	Low	Low	Low	Low	Moderate	Low
Chevalier et al. [23]	Moderate	Low	Low	Low	Moderate	Moderate
Conrozier et al. [25]	Moderate	Low	Low	Low	Low	Low
Conrozier et al. [26]	Moderate	Low	Low	Low	Low	Low
Danielsson [27, 28]	Low	High	High	High	High	High
van Dijk et al. [29]	*Low*	*Low*	*Low*	*Low*	*Low*	*Low*
van Dijk et al. [30]	*Low*	*Low*	*Low*	*Low*	*Low*	*Low*
Dorleijn 2015 [31]	Low	Low	Moderate	Low	Moderate	Low
Dougados et al. [32]	*Low*	*Low*	*Low*	*Low*	*Low*	*Low*
Dougados et al. [33]	Low	Low	Low	Moderate	High	Moderate
Dougados et al. [34]	*Low*	*Low*	*Low*	*Low*	*Low*	*Low*
Fukushima et al. [35]	Moderate	Low	Low	High	High	Low
Golightly et al. [36]	Low	Moderate	Low	Low	Low	Low
Gossec et al. [37]	*Low*	*Low*	*Low*	*Low*	*Low*	*Low*
Hartofilakidis et al. [38]	Moderate	Moderate	Moderate	Moderate	High	High
Hawker et al. [39]	Moderate	Low	Low	Low	Low	Low
Hoeven et a. [40]	Low	Moderate	Low	Low	Low	Low
Holla et al. [41]	*Low*	*Low*	*Low*	*Low*	*Low*	*Low*
Juhakoski et al. [42]	Low	Low	Low	Moderate	Low	Low
Kalyoncu et al. [43]	Low	Low	Moderate	Moderate	Low	Low
Kelman et al. [44]	*Low*	*Low*	*Low*	*Low*	*Low*	*Low*
Kerkhof et al. [45]	Low	Moderate	Moderate	Low	Low	Low
Kopec et al. [46]	Low	Moderate	Low	Low	Low	Low
Lane et al. [47]	*Low*	*Low*	*Low*	*Low*	*Low*	*Low*
Lane et al. [48]	Moderate	Low	Moderate	Low	Low	Low
Laslett et al. [49]	*Low*	*Low*	*Low*	*Low*	*Low*	*Low*
Ledingham et al. [50]	Moderate	Moderate	Moderate	High	High	High
Lievense et al. [51]	Low	Low	Moderate	Low	Low	Low
Maillefert et al. [52]	Low	Low	Low	Moderate	Moderate	Moderate
Mazieres et al. [53]	*Low*	*Low*	*Low*	*Low*	*Low*	*Low*
Nelson et al. [54]	Low	Moderate	Low	Low	Low	Low
Peters et al. [56]	Low	Moderate	Moderate	Low	Moderate	Low
Pisters et al. [57]	*Low*	*Low*	*Low*	*Low*	*Low*	*Low*
Pollard et al. [58]	Low	Low	Low	Moderate	Low	Low
Reijman et al. [59]	Low	Moderate	Low	Low	Low	Low
Reijman et al. [60]	Low	Moderate	Low	Low	Low	Low
Reijman et al. [61]	Low	Moderate	Low	Low	Low	Low
Solignac [62]	Low	Low	Low	Low	Moderate	Low
van Spil et al. [63]	*Low*	*Low*	*Low*	*Low*	*Low*	*Low*
Tron et al. [65]	High	High	High	Moderate	High	Moderate
Verkleij et al. [66]	Low	Low	Low	Low	Moderate	Low
Vinciguerra et al. [67]	Low	Moderate	High	Low	High	High

Studies with a low risk of bias in all domains are presented in italics

### Prognostic factors

We identified 154 possible prognostic factors: 23 patient variables, 77 disease characteristics, and 54 chemical markers or imaging markers. Fifty-one factors were only investigated once in a single cohort or study (not a low risk of bias study) and could not be included in the evidence synthesis. An overview of all the results and risk of bias assessment of the studies describing these factors is presented in Additional file 4. The remaining 103 factors were included in the evidence synthesis. To decrease heterogeneity, evidence synthesis was done separately per group of outcomes (radiological progression, clinical progression, or THR). However, heterogeneity was still considered high in each outcome group, mainly within respect to the definition of the prognostic factor, progression, and measure of the association. Therefore, we refrained from pooling and performed a best-evidence synthesis. If a factor could not be subdivided because it was described by two or three studies that used a definition of progression, all in a separate group of outcome, we combined the groups of outcomes. The results of these factors are presented in Additional file 5.

### Evidence for factors predicting progression

Strong evidence was found for a higher K-L grade at baseline, superior or (supero) lateral femoral head migration, and subchondral sclerosis to be predictive of faster progression to THR or more patients progressing to THR. Body mass index was found not to be predictive of faster or more progression to THR (Table 3).

**Table 3:** Factors predicting (indication for) total hip replacement (THR)

Prognostic factor	Studies	Associations	Best-evidence synthesis
Patient variables			
No association			
Body mass index			Strong evidence for no association
	2 low risk of bias cohorts [16, 37] 5 cohorts [20, 39, 50, 51, 67]	No, no No, no, no, negative, positive	
Female			Moderate evidence for no association
	3 low risk of bias cohorts [16, 34, 37] 5 cohorts [20, 39, 50–52]	No, positive, no No, no, no, no, no	
Lower educational level			Moderate evidence for no association
	1 low risk of bias cohort [16] 1 cohort [39]	No No	
Western or White ethnicity			Moderate evidence for no association
	1 low risk of bias cohort [16] 1 cohort [39]	No No	
Alcohol consumption			Limited evidence for no association
	1 low risk of bias cohort [16]	No	
Conflicting evidence			
Higher age at baseline			Conflicting evidence
	3 low risk of bias cohorts [16, 34, 37] 5 cohorts [20, 39, 50, 51, 67]	No, positive,no No, positive^$^, no, no, positive	
Disease characteristics			
Faster or more progression			
Lower global assessment (self-reported) at baseline			Moderate evidence for faster or more progression
	1 low risk of bias cohort [37] 2 cohorts [39, 50]	Positive Positive, positive	
Previous use of NSAIDs			Limited evidence for more progression
	1 low risk of bias cohort [37]	Positive	
No association			
Longer duration of symptoms at baseline			Moderate evidence for no association
	1 low risk of bias cohort [37] 1 cohort [19]	No No	
Having another disease (comorbidity)			Moderate evidence for no association
	1 low risk of bias cohort [16] 1 cohort [39]	No No	
Morning stiffness			Moderate evidence for no association
	1 low risk of bias cohort [16] 1 cohort [51]	No No	
Use of pain medication at baseline			Moderate evidence for no association
	1 low risk of bias cohort [16] 1 cohort [19]	No No	
Presence of Heberden’s or Bouchard’s nodes			Moderate evidence for no association
	1 low risk of bias cohort [16] 2 cohorts [50, 51]	No No, no	
Previous intra-articular injection in the hip			Limited evidence for no association
	1 low risk of bias cohort [37]	No	
Conflicting evidence			
More limitations in physical function at baseline			Conflicting evidence
	3 low risk of bias cohorts [16, 34, 37] 2 cohorts [19, 39]	Positive, positive, no No, no	
More pain at baseline			Conflicting evidence
	3 low risk of bias cohorts [16, 34, 37] 4 cohorts [19, 39, 50, 51]	Conflicted^$$^, positive, positive Positive, no, positive, no	
Painful hip flexion (active or passive)			Conflicting evidence
	1 low risk of bias cohort [16] 1 cohort [51]	Positive No	
Painful hip internal rotation (active or passive)			Conflicting evidence
	1 low risk of bias cohort [16] 1 cohort [51]	Positive No	
Night pain at baseline			Conflicting evidence
	2 cohorts [50, 51]	Positive, no	
Limited range of motion of flexion of the hip			Conflicting evidence
	1 low risk of bias cohort [16] 2 cohorts [19, 51]	Positive Positive, no	
Limited range of motion of internal hip rotation			Conflicting evidence
	1 low risk of bias cohort [16] 2 cohorts [19, 51]	Positive Positive, no	
Limited range of motion of external hip rotation			Conflicting evidence
	2 cohorts [19, 51]	Positive, no	
Chemical or imaging markers			
Faster or more progression			
Higher K-L grade at baseline			Strong evidence for more or faster progression
	2 low risk of bias cohorts [34, 37] 1 cohorts [51]	Positive, positive Positive	
Superior or superolateral migration of the femoral head			Strong evidence for more or faster progression
	2 low risk of bias cohorts [34, 47] 1 cohort [38]	Positive, positive Positive	
Subchondral sclerosis			Strong evidence for more progression
	2 low risk of bias cohorts [16, 47]	Positive, positive	
Statistical shape modeling			Moderate evidence that certain modes of SSM can predict progression
	3 cohorts [11, 12, 12]	Positive, positive, positive	
Joint space narrowing at baseline			Moderate evidence for more or faster progression
	1 low risk of bias cohort [16] 1 cohort [67]	Positive Positive	
No association			
Cam-type deformity (alpha angle > 60°)			Limited evidence for no association
	1 low risk of bias cohort [16]	No	
Conflicting evidence			
Erythrocyte sedimentation rate			Conflicting evidence
	1 low risk of bias cohort [16] 1 cohort [51]	Positive No	
Atrophic bone response (no osteophytes present)			Conflicting evidence
	1 low risk of bias cohort [16] 2 cohorts [50, 51]	Positive Positive, no	
Decrease in joint space width at baseline			Conflicting evidence
	1 low risk of bias cohort [34] 1 cohort [51]	Positive No	
Wiberg’s center edge angle (CEA)			Conflicting evidence
	1 low risk of bias cohort [16] 1 cohort [20]	Negative No	

^$^Exception: age ≥ 82 years showed a negative association with progression, compared to age ≤ 62 years

^$$^Pain at baseline measured with NRS past week showed a statistically significant positive association with THR; pain at baseline measured with WOMAC pain showed no statistically significant association with THR

Strong evidence was found for no association between radiological progression and the following markers: C-terminal telopeptide of collagen type I (CTX-I), cartilage oligomeric matrix protein (COMP), N-terminal telopeptide of collagen type I (NTX-I), and N-terminal propeptide of procollagen type I and type III (PINP, PIIINP) (Table 4).

**Table 4:** Factors predicting radiological progression

Prognostic factor	Studies	Associations	Best-evidence synthesis
Patient variables			
No association			
Family history of OA			Moderate evidence for no association
	3 cohorts [25, 60, 65]	No, no, no	
Body mass index			Moderate evidence for no association
	4 cohorts [25, 50, 61, 65]	No, no, no, no	
Conflicting evidence			
Higher age at baseline or at first symptoms			Conflicting evidence
	1 low risk of bias cohort [32] 4 cohorts [35, 50, 60, 65]	Positive No, positive, positive, no	
Female			Conflicting evidence
	1 low risk of bias cohort [32] 6 cohorts [25, 27, 35, 50, 60, 65]	Positive No, no, no, no, positive, no	
Disease characteristics			
Faster or more progression			
More limitations in physical function at baseline			Moderate evidence for more progression
	1 low risk of bias cohort [32] 1 cohort [60]	Positive Positive	
Hip pain present at baseline or on most days for a least 1 month in the past year			Moderate evidence for more progression
	1 low risk of bias cohort [47] 1 cohort [60]	Positive Positive	
No association			
Forestier’s disease			Moderate evidence for no association
	3 cohorts [25, 50, 65]	No, no, no	
Diabetes mellitus			Limited evidence for no association
	2 cohorts [25, 60]	No, no	
Bilateral hip OA			Limited evidence for no association
	2 cohorts [25, 65]	No, no	
Generalized OA			Limited evidence for no association
	2 cohorts [25, 65]	No, no	
Chemical or imaging markers			
Faster or more progression			
Subchondral sclerosis			Moderate evidence for more progression
	1 low risk of bias cohort [47] 1 cohort [33]	Positive Positive	
Neck width of the femoral head			Limited evidence for more progression
	1 low risk of bias cohort [21]	Positive	
Osteocalcin (OC)			Limited evidence for less progression
	1 low risk of bias cohort [63]	Negative	
No association			
C-terminal telopeptide of collagen type I (CTX-I)			Strong evidence for no association
	2 low risk of bias cohorts [53, 63]	No, no	
Cartilage oligomeric matrix protein (COMP)			Strong evidence for no association
	3 low risk of bias cohorts [44, 53, 63] 1 cohort [26]	No, no, no Positive	
N-terminal telopeptide of collagen type I (NTX-I)			Strong evidence for no association
	2 low risk of bias cohorts [44, 63]	No, no	
N-terminal propeptide of procollagen type I (PINP)			Strong evidence for no association
	2 low risk of bias cohorts [53, 63]	No, no	
N-terminal propeptide of procollagen type III (PIIINP)			Strong evidence for no association
	2 low risk of bias cohorts [53, 63]	No, no	
High-sensitive C-reactive protein (hs-CRP)			Moderate evidence for no association
	1 low risk of bias cohort [53] 1 cohort [45]	No No	
Angle of the femoral head			Moderate evidence for no association
	1 low risk of bias cohort [21] 2 cohorts [20, 65]	No No, no	
Acetabular osteophytes only			Moderate evidence for no association
	1 low risk of bias cohort [47] 1 cohort [33]	No No	
N-terminal propeptide of procollagen type IIA (PIIANP)			Limited evidence for no association
	1 low risk of bias cohort [63]	No	
Chondroitin sulphate 846 (CS846)			Limited evidence for no association
	1 low risk of bias cohort [63]	No	
Cartilage glycoprotein 40 (YKL-40)			Limited evidence for no association
	1 low risk of bias cohort [53]	No	
Matrix metalloproteinases (MMP-1)			Limited evidence for no association
	1 low risk of bias cohort [53]	No	
Matrix metalloproteinases (MMP-3)			Limited evidence for no association
	1 low risk of bias cohort [53]	No	
Neck length of the femoral head			Limited evidence for no association
	1 low risk of bias cohort [21]	No	
Conflicting evidence			
Bone mineral content			Conflicting evidence
	1 low risk of bias cohort [21]	Conflicted^$^	
Area/size of the hip joint			Conflicting evidence
	1 low risk of bias cohort [21]	Conflicted^$$^	
C-terminal telopeptide of collagen type II (CTX-II)			Conflicting evidence
	2 low risk of bias cohorts [53, 63] 1 cohort [59]	Positive, no Positive	
Hyaluronic acid (HA)			Conflicting evidence
	2 low risk of bias cohorts [53, 63] 1 cohort [23]	Positive, no No	
Atrophic bone response (no osteophytes present)			Conflicting evidence
	1 low risk of bias cohort [47] 3 cohorts [25, 50, 65]	No Positive, positive, no	
Subchondral cysts			Conflicting evidence
	1 low risk of bias cohort [47] 1 cohort [33]	Positive No	
Decrease in joint space width at baseline			Conflicting evidence
	1 low risk of bias cohort [32] 2 cohorts [25, 60]	Positive No, positive	
Superior or (supero) lateral migration of the femoral head			Conflicting evidence
	2 low risk of bias cohorts [32, 47] 2 cohorts [25, 50]	Positive, no No, positive	
Higher K-L grade at baseline			Conflicting evidence
	4 cohorts [33, 50, 60, 65]	No, positive, positive, no	
Acetabular index (Horizontal toit externe angle)			Conflicting evidence
	2 cohorts [20, 65]	Conflicted^$$$^, no	
Wiberg’s center edge angle (CEA)			Conflicting evidence
	2 cohorts [20, 65]	No, negative	

^$^BMC of superior (*p* = 0.009) and medial (*p* = 0.019) quart femoral head, arc regions 2–4 (*p* = 0.02, 0.001, 0.003, respectively), and the acetabular arc was higher in patients with progression than without progression. BMC of the femoral neck (*p* = 0.17), intertrochanteric area (*p* = 0.9), trochanteric area (*p* = 0.6), and inferior (*p* = 0.08) and lateral (*p* = 0.06) quart femoral head and arc region 1 (*p* = 0.19) of acetabular arc was not significantly different between patients with or without progression

^$$^The area/size of superior (*p* = 0.002), medial (p = 0.002), inferior (*p* = 0.003), and lateral (*p* = 0.003) femoral head and of arc regions 2–4 (*p* = 0.007, 0.001 and 0.005 respectively) of acetabular arc was higher in patients with progression than without progression. The area/size of the femoral neck (*p* = 0.6), intertrochanteric area (*p* = 0.16), trochanteric area (*p* = 0.4), and arc region 1 (*p* = 0.2) of the acetabular arc was not significantly different between patients with progression and without progression.

^$$$^A statistically significant association was found between the acetabular index and progression defined as ≥ 1 increase in joint space narrowing; however, no statistically significant association was found between the acetabular index and progression defined as ≥ 1 increase in K-L grade

Strong evidence showed comorbidity to be predictive of clinical progression. On the other hand, gender, social support, use of pain medication at baseline, quality of life at baseline, and limited range of motion of internal hip rotation or external hip rotation were not predictive of clinical progression (Table 5).

**Table 5:** Factors predicting clinical progression

Prognostic factor	Studies	Associations	Best-evidence synthesis
Patient variables			
No association			
Female			Strong evidence for no association
	2 low risk of bias cohorts [41, 57] 5 cohorts [13, 27, 42, 56, 66]	No, no Positive, no, no, no, no	
Social support			Strong evidence for no association
	2 low risk of bias cohorts [41, 57]	No, no	
Higher age at baseline			Moderate evidence for no association
	1 low risk of bias cohort [41, 57] 3 cohorts [42, 56, 66]	No, positive No, no, no	
Paid employment			Moderate evidence for no association
	1 low risk of bias cohort [41] 2 cohorts [42, 56]	No No, no	
Living alone			Moderate evidence for no association
	1 low risk of bias cohort [41] 1 cohort [30]	No No	
Alcohol consumption			Limited evidence for no association
	1 low risk of bias cohort [41]	No	
Conflicting evidence			
Physical activity during leisure			Conflicting evidence
	1 low risk of bias cohort [41]	Conflicted^$^	
Body mass index			Conflicting evidence
	2 low risk of bias cohorts [41, 57] 3 cohorts [42, 56, 66]	Positive, no No, no, positive	
Lower education level			Conflicting evidence
	2 low risk of bias cohorts [41, 57] 2 cohorts [42, 66]	No, negative Positive, no	
Disease characteristics			
Faster or more progression			
Having another disease (comorbidity)			Strong evidence for more progression
	2 low risk of bias cohorts [41, 57] 1 cohort [42]	Positive^$$^, positive Positive	
Concurrent morning stiffness of the knee (< 30 min)			Limited evidence for more progression
	1 low risk of bias cohort [41]	Positive	
No association			
Use of (pain) medication at baseline			Strong evidence for no association
	2 low risk of bias cohorts [29, 41]	No, no	
Quality of life at baseline			Strong evidence for no association
	2 low risk of bias cohort [30, 41]	No^$$$^, no	
Limited range of motion of internal hip rotation			Strong evidence for no association
	2 low risk of bias cohorts [41, 57] 1 cohort [66]	No, no No	
Limited range of motion of external hip rotation			Strong evidence for no association
	2 low risk of bias cohorts [15, 57]	No, no	
Concurrent knee pain			Moderate evidence for no association
	1 low risk of bias cohort [41] 1 cohort [66]	No No	
Depression			Moderate evidence for no association
	1 low risk of bias cohort [41] 1 cohort [56]	No No	
Way of coping			Moderate evidence for no association
	1 low risk of bias cohort [41] 1 cohort [30]	No No	
Respiratory comorbidity			Moderate evidence for no association
	1 low risk of bias cohort [29] 1 cohort [56]	No No	
Patient-rated health			Limited evidence for no association
	1 low risk of bias cohort [41]	No	
Cardiac comorbidity (cumulative illness rating scale 1, severity score ≥ 2)			Limited evidence for no association
	1 low risk of bias cohort [29]	No	
Vascular comorbidity (cumulative illness rating scale 2, severity score ≥ 2)			Limited evidence for no association
	1 low risk of bias cohort [29]	No	
Eye, ear, nose, throat, and larynx diseases (cumulative illness rating scale 4, severity score ≥ 2)			Limited evidence for no association
	1 low risk of bias cohort [29]	No	
Upper gastrointestinal comorbidity (cumulative illness rating scale 5, severity score ≥ 2)			Limited evidence for no association
	1 low risk of bias cohort [29]	No	
Lower gastrointestinal comorbidity (cumulative illness rating scale 6, severity score ≥ 2)			Limited evidence for no association
	1 low risk of bias cohort [29]	No	
Hepatic comorbidity (cumulative illness rating scale 7, severity score ≥ 2)			Limited evidence for no association
	1 low risk of bias cohort [29]	No	
Renal comorbidity (cumulative illness rating scale 8, severity score ≥ 2)			Limited evidence for no association
	1 low risk of bias cohort [29]	No	
Other genitourinary comorbidities (cumulative illness rating scale 9, severity score ≥ 2)			Limited evidence for no association
	1 low risk of bias cohort [29]	No	
Neurological comorbidity (cumulative illness rating scale 11, severity score ≥ 2)			Limited evidence for no association
	1 low risk of bias cohort [29]	No	
Psychiatric comorbidity (cumulative illness rating scale 12, severity score ≥ 2)			Limited evidence for no association
	1 low risk of bias cohort [29]	No	
Comorbidity of endocrine and metabolic diseases (cumulative illness rating scale 13, severity score ≥ 2)			Limited evidence for no association
	1 low risk of bias cohort [29]	No	
Cognitive functioning			Limited evidence for no association
	1 low risk of bias cohort [57]	No	
Muscle strength hip abduction			Limited evidence for no association
	1 low risk of bias cohort [57]	No	
Pain during sitting or lying			Limited evidence for no association
	1 low risk of bias cohort [41]	No	
Joint stiffness (WOMAC)			Limited evidence for no association
	1 low risk of bias cohort [15]	No	
Use of additional supplements or vitamins			Limited evidence for no association
	1 low risk of bias cohort [15]	No	
Concurrent pain during flexion of ipsilateral knee			Limited evidence for no association
	1 low risk of bias cohort [15]	No	
Knee flexion			Limited evidence for no association
	1 low risk of bias cohort [29]	No	
Knee extension			Limited evidence for no association
	1 low risk of bias cohort [29]	No	
Strength of isometric knee extension			Limited evidence for no association
	1 low risk of bias cohort [29]	No	
Conflicting evidence			
Bilateral hip OA			Conflicting evidence
	1 low risk of bias cohort [41] 1 cohort [66]	Positive, if equal symptoms No	
Pain at baseline (self-reported or during physical examination)			Conflicting evidence
	3 low risk of bias cohorts [29, 41, 47]	No, no, positive	
Longer duration of symptoms at baseline			Conflicting evidence
	1 low risk of bias cohort [57] 2 cohorts [42, 66]	No No, positive	
Morning stiffness			Conflicting evidence
	1 low risk of bias cohort [41] 1 cohort [66]	No Positive	
Limited range of motion of flexion of the hip			Conflicting evidence
	2 low risk of bias cohorts [41, 57] 1 cohort [66]	Positive, no No	
Chemical or imaging markers			
Conflicting evidence			
Higher K-L grade at baseline			Conflicting evidence
	1 low risk of bias cohort [12] 2 cohorts [42, 66]	No No, positive	

^$^Patients who were 3–5 days/week physically active in their leisure time showed less progression than patients who were 0–2 days/week physically active in their leisure time. No difference was found between patients spending 6–7 days/week on physical activity and patients spending 0–2 days/week on physical activity

^$$^≥ 3 more diseases compared to no comorbidities

^$$$^Subscale of SF-36 vitality showed a positive association with WOMAC function score

For other factors, only moderate, limited, or conflicting evidence was found for predicting or not predicting progression (Tables 3, 4, and 5).

## Discussion

In this study, we systematically reviewed all 154 factors predictive of progression of hip OA, reported in 57 studies. Compared to earlier reviews, there was a considerable amount of additional evidence available for the factors previously reported in reviews, as well as evidence for factors not earlier described.

In this review, some results had changed compared to the review of Lievense et al. in 2002 [2]. Firstly, because of the new evidence emerging from the later studies, especially studies with a clinical outcome of progression. Secondly, because we used a different method to assess the risk of bias, some studies were no longer considered to have a low risk of bias. The QUIPS tool seems to apply stricter criteria than the method used by Lievense et al. in 2002. Thirdly, we divided the outcomes into three different groups of progression. Thus, due to these methodological differences (together with additional studies), we were unable to confirm an atrophic bone response as a predictor for radiological progression or progression to THR. On the other, we were able to confirm their conclusion on BMI as not predictive of progression and faster progression in patients with a superolateral migration of the femoral head.

Most of the prognostic factors reported by Wright et al. in 2009 [3] were confirmed in this present review in one or more of the outcome groups. The differences found in age, femoral and acetabular osteophytes, and hip pain at baseline were (as with Lievense et al.) a combination of new evidence, differences in the risk of bias assessment, and the division into defined groups of progression. The study from de Rooij et al. in 2016 [4] reviewed the evidence for predictors of the course of pain and function and found comorbidity and vitality (SF-36) to be predictive of function, as we found for clinical progression. However, although they also used the QUIPS tool to assess the risk of bias, they used a different cutoff point to classify a study as having a low risk of bias. Therefore, some earlier findings of strong evidence for no association with the course of pain or function were confirmed as only moderate evidence for no association with clinical progression in our review. Other differences between this review and the present one are mainly attributable to the differences in the selection criteria. In Table 6, we summarized all factors with strong evidence to be predictive of progression found in one of these four reviews and the overlap and differences in evidence for these factors.

**Table 6:** Overview of factors with strong evidence to be predictive of progression, overlap and differences between this review and the review of de Rooij et al., Wright et al., and Lievense et al.

Prognostic factor	Teirlinck et al. factor predictive of	De Rooij et al. factor predictive of	Wright et al. factor predictive of	Lievense et al. factor predictive of
K-L grade at baseline	**THR**	Strong evidence for no association for clinical progression	Radiological progression or THR*	Not mentioned
Subchondral sclerosis at baseline	**THR**	Not mentioned	**Radiological progression and/or THR**	Not mentioned
Superior or (supero) lateral femoral head migration	**THR**	Not mentioned	**Radiological progression and/or THR**	**Radiological progression and/or THR**
Comorbidity	**Clinical progression**	**Clinical progression (strong evidence for a course of function, weak evidence for a course of pain)**	Not mentioned	Not mentioned
Low vitality	Quality of life in general: strong evidence of no association, specific for SF 36 vitality: strong evidence for clinical progression	**Course of function**	Not mentioned	Not mentioned
Age	Conflicted evidence for THR and radiological progression, moderate evidence for no association with clinical progression	Strong evidence for no association with pain and conflicted evidence for function	**Radiological progression and/or THR**	Conflicted evidence
Femoral osteophytes	Conflicted evidence	Not mentioned	**Radiological progression and/or THR**	Not mentioned
Hip pain at baseline	Conflicted evidence	Conflicted evidence	**Radiological progression and THR**	Not mentioned
JSW at baseline	Conflicted evidence	Not mentioned	**Radiological progression and/or THR**	Limited evidence for THR
Lequesne index score ≥ 10 at baseline	Conflicted evidence for THR, moderate evidence for radiological progression**	Conflicted evidence**	**Radiological progression and/or THR**	Not mentioned
Atrophic bone response	Conflicted evidence	Not mentioned	Conflicted evidence	**Radiological progression**

*K-L grade 3 at baseline

**Function at baseline in general

bold text represents strong evidence to be predictive of progression

Strengths of this present review are the sensitive literature search and our systematic approach to the selection, risk of bias assessment, and the best-evidence synthesis. Therefore, we have presented an extensive overview of reported prognostic factors and existing evidence for their associations. In performing the evidence synthesis divided into outcome (radiological, clinical, or THR), we decreased the heterogeneity and we believe the results to be more accurate for daily practice. Unfortunately, heterogeneity was still too high to perform a meta-analysis. Therefore, we were bound to a best-evidence synthesis and unable to calculate the strengths of the associations. This limits the translation to the daily clinical practice. Another disadvantage of this synthesis compared to a meta-analysis is that smaller studies contribute to the result with the same weight as larger studies, even though the smaller studies may have low power to show a statistically significant association.

In the selection of studies, several restrictions were imposed. First, languages were restricted to ensure that at least two researchers had a reasonable understanding of the languages included so all articles were reliably assessed. However, this implies that we may have missed studies from countries in which publication in English is less common. Secondly, negative results (i.e., no association was found) are less likely to be published and are therefore not well represented in this review.

We used the QUIPS tool to assess the risk of bias. Nine other studies using this tool reported an inter-rater agreement ranging from 70 to 89.5% (median 83.5%) and a kappa statistic ranging from 0.56 to 0.82 (median 0.75) [7]. Compared to these data, our inter-rater agreement was low and considered to be moderate. Disagreement was mainly due to the differences in interpretation of items of the QUIPS tool; however, only for very few items, a third reviewer was needed to make a final decision.

Hip dysplasia and femoral acetabular impingement were initially considered to be underlying pathologies and were excluded from this analysis. However, the range of severity of these morphologies is substantial, i.e., some of these morphologies should clearly be considered as an underlying pathology, whereas others are more subtle and sometimes undiagnosed. These subtle morphologies might be considered to be possible prognostic factors, rather than underlying pathologies. Therefore, all citations were screened using the terms “hip dysplasia” and “femoral acetabular impingement” in the title or abstract. However, we found only one small study [35] which investigated the radiographic findings of femoral acetabular impingement as a prognostic factor (results of this study are included in Additional file 4). In the studies already included, three studies did not specifically include patients with hip dysplasia or femoral acetabular impingement but did investigate the associated angles (Wiberg’s center edge angle and alpha angle, respectively). Since the evidence for these associations with the progression of hip OA was weak, future studies and reviews should investigate these morphologies as possible prognostic factors.

## Conclusion

We conclude that there is consistent evidence that four factors (comorbidity, K-L grade, superior or (supero) lateral femoral head migration, and subchondral sclerosis) were predictive of progression of hip OA, whereas 12 factors were not predictive. The evidence for other factors was weak or conflicting. Health professionals caring for patients with hip OA will benefit from the insight in prognostic factors, e.g., patients more likely to progress rapidly may need an intensified symptomatic treatment or early referral to an orthopedic surgeon. For this, we still need more high-quality research focusing on the prognostic factors in hip OA.

## Additional files

Additional file 1: Syntax of literature search. (DOCX 15 kb)

Additional file 2: Criteria items of QUIPS tool and possible adjustments. (DOCX 42 kb)

Additional file 3: Characteristics of the selected studies: extensive overview. (DOCX 172 kb)

Additional file 4: Prognostic factors described by one study or multiple studies from the same cohort. (DOCX 126 kb)

Additional file 5: Factors predicting total hip replacement, clinical or radiological progression combined. (DOCX 82 kb)

## Data Availability

Data sharing is not applicable to this article as no datasets were generated or analyzed during the current study.

## Abbreviations

BMI: Body mass index
COMP: Cartilage oligomeric matrix protein
CS846: Chondroitin sulphate 846
CTX-I: C-terminal telopeptide of collagen type I
CTX-III: C-terminal telopeptide of collagen type II
ESR: Erythrocyte sedimentation rate
GRADE: Grading of Recommendations Assessment, Development and Evaluation
HA: Hyaluronic acid
hs-CRP: High-sensitive C-reactive protein
JSN: Joint space narrowing
JSW: Joint space width
K-L grade: Kellgren and Lawrence grade
LCGA: Latent class growth analysis
MJS: Minimum joint space
MMP-1: Matrix metalloproteinases-1
MMP-3: Matrix metalloproteinases-3
MRI: Magnetic resonance imaging
NRS: Numeric rating scale
NTX-I: N-terminal telopeptide of collagen type I
OA: Osteoarthritis
OC: Osteocalcin
PIIANP: N-terminal propeptide of procollagen type IIA
PIIINP: N-terminal propeptide of procollagen type III
PINP: N-terminal propeptide of procollagen type I
QUIPS: Quality in prognosis studies
THR: Total hip replacement
VAS: Visual analog scale
WOMAC: Western Ontario and McMaster Universities Osteoarthritis Index
YKL-40: Cartilage glycoprotein 40
YMN: Yearly mean narrowing
