# Supplementary material for: Prognostic factors for progression of osteoarthritis of the hip: a systematic review
Source: Arthritis Res Ther. 2019 Aug 23;21:192. doi: 10.1186/s13075-019-1969-9 (PMC6708123; doi:10.1186/s13075-019-1969-9)
Supplement: Supplementary file 1 — Syntax of literature search. (DOCX 15 kb) [file 13075_2019_1969_MOESM1_ESM.docx]

Additional file 1 Syntax of literature search

**Embase.com**

('hip osteoarthritis'/de OR 'coxitis'/de OR ('osteoarthritis'/de AND 'hip'/de) OR (((hip OR cox OR 'femoral head' OR 'femur head' OR 'lower extremity' OR 'lower extremities') NEAR/6 (osteoarth* OR arthrit* OR artherosis* OR artherotic* OR arthrosis* OR arthrotic* OR 'OA')) OR coxitis OR coxarth* OR 'malum coxae senilis'):ab,ti) AND ('disease course'/de OR 'disease severity'/de OR 'prognosis'/de OR prediction/de OR 'deterioration'/de OR (((disease* OR clinical OR illness* OR pain* OR function*) NEAR/6 (course* OR development* OR evolut* OR sever*)) OR prognos* OR deteriorat* OR progressi* OR declin* OR predict* OR precipitat* OR exacerbat*):ab,ti) NOT ([Conference Abstract]/lim OR [Conference Paper]/lim OR [Letter]/lim OR [Editorial]/lim OR [Note]/lim OR [Conference Review]/lim)

**Medline (OvidSP)**

("Osteoarthritis, Hip"/ OR ("osteoarthritis"/ AND ("hip"/ OR "hip joint"/)) OR (((hip OR cox OR "femoral head" OR "femur head" OR "lower extremity" OR "lower extremities") ADJ6 (osteoarth* OR arthrit* OR artherosis* OR artherotic* OR arthrosis* OR arthrotic* OR "OA")) OR coxitis OR coxarth* OR "malum coxae senilis").ab,ti.) AND ("prognosis"/ OR "Disease Progression"/ OR (((disease* OR clinical OR illness* OR pain* OR function*) ADJ6 (course* OR development* OR evolut* OR sever*)) OR prognos* OR deteriorat* OR progressi* OR declin* OR predict* OR precipitat* OR exacerbat*).ab,ti.) NOT (Congresses OR comment OR Letter OR Editorial OR news OR Published Erratum).pt.

**Cochrane**

((((hip OR cox OR 'femoral head' OR 'femur head' OR 'lower extremity' OR 'lower extremities') NEAR/6 (osteoarth* OR arthrit* OR artherosis* OR artherotic* OR arthrosis* OR arthrotic* OR 'OA')) OR coxitis OR coxarth* OR 'malum coxae senilis'):ab,ti) AND ((((disease* OR clinical OR illness* OR pain* OR function*) NEAR/6 (course* OR development* OR evolut* OR sever*)) OR prognos* OR deteriorat* OR progressi* OR declin* OR predict* OR precipitat* OR exacerbat*):ab,ti)

**Web-of-science**

TS=(((((hip OR cox OR "femoral head" OR "femur head" OR "lower extremity" OR "lower extremities") NEAR/6 (osteoarth* OR arthrit* OR artherosis* OR artherotic* OR arthrosis* OR arthrotic* OR "OA")) OR coxitis OR coxarth* OR "malum coxae senilis")) AND ((((disease* OR clinical OR illness* OR pain* OR function*) NEAR/6 (course* OR development* OR evolut* OR sever*)) OR prognos* OR deteriorat* OR progressi* OR declin* OR predict* OR precipitat* OR exacerbat*))) AND DT=(article)

**Google Scholar**

"hip|cox osteoarthritis|arthritis|artherosis|arthrosis|OA"|coxitis|coxarthrosis "disease|clinical|pain|functional course|development|severity"|prognosis|prognostic|deterioration|progression|decline|prediction
