# Supplementary material for: Prognostic factors for progression of osteoarthritis of the hip: a systematic review
Source: Arthritis Res Ther. 2019 Aug 23;21:192. doi: 10.1186/s13075-019-1969-9 (PMC6708123; doi:10.1186/s13075-019-1969-9)
Supplement: Supplementary file 3 — Characteristics of the selected studies: extensive overview. (DOCX 172 kb) [file 13075_2019_1969_MOESM3_ESM.docx]

Additional file 3. Characteristics of the selected studies: extensive overview

| Study | Design | Setting | Study population | Participants in cohort (n) | Age (years) ± SD | Female (%) | Recruitment | Assessment of progression | Follow-up |
| --- | --- | --- | --- | --- | --- | --- | --- | --- | --- |
| Agricola 2013[[1](#_ENREF_1)] | Prospective cohort  (CHECK) | Combination of primary, secondary care and population-based (NED) | Patients and participants, 45-65 years, pain and/or stiffness in 1 or both knees and/or hips, K-L grade =1 | 1002 (analyzed 723 patients with AP pelvis radiographs) | 55.9 ±5.2 | 79.5 | 2002-2005 | THR | 5 years |
| Agricola 2015a[[2](#_ENREF_2)] | Prospective cohort  (CHECK) | Combination of primary, secondary care and population-based (NED) | Patients and participants, women, 45-65 years, pain and/or stiffness in 1 or both knees and/or hips | 1002 (analyzed 550 women) | 55.8 ±5.1 | 100 | 2002-2005 | THR due to OA | 5 years |
| Agricola 2015b[[2](#_ENREF_2)] | Nested case-control (Chingford cohort) | Population-based, from 1 general practice (UK) | Participants, women, 44-67 years, K-L grade <2 | 1003 (analyzed 114) | 53.6 ±5.4 | 100 | 1989 | THR due to OA | 19 years |
| Auquier 1979[[3](#_ENREF_3)] | Retrospective cohort | Secondary care, Internal medicine and Rheumatology (FRA) | Patients with primary hip OA, ≥ 6 years between first and last examination, ≥ 4 examinations | 131 | 52.18±10.21 | 48 | 1948-1965 | Increase in stage of pain and function, stages minimal, moderate, moderate-severe, severe | 6-23 years |
| Barr 2012[[4](#_ENREF_4)] | Case-control | Primary care (UK) | Patients, with a new episode of hip pain, not thought to originate from structures outside the hip, age ≥40 years | 195 (analyzed 102 patients) | 62.7±10.7 | 68 | NR | THR (compared to non-progression hips: increase of ≤ 1 K-L grade) | 5 years |
| Bastick 2016[[5](#_ENREF_5)] | Prospective cohort (CHECK) | Combination of primary, secondary care and population-based (NED) | Patients and participants, 45-65 years, pain and/or stiffness in 1 or both knees and/or hips | 545 (analyzed 363 patients) | 55.7±5.2 | 81 | 2002-2005 | NRS score for pain, group moderate progression compared to mild pain. Groups based on LCGA. | 5 years |
| Bastick 2017[[6](#_ENREF_6)] | Prospective cohort (CHECK) | Combination of primary, secondary care and population-based (NED) | Patients and participants, 45-65 years, pain and/or stiffness in 1 or both knees and/or hips | 588 (analyzed 538 patients) | 55.7±5.3 | 79 | 2002-2005 | THR | 5 years |
| Bergink 2016[[7](#_ENREF_7)] | Prospective cohort (Rotterdam I) | Population-based (NED) | Participants ≥ 55 years, radiographs of hips available and scored at baseline and follow-up | 176 | NR | NR | 1989 - 1993 | 1. Increase ≥1 K-L grade  2. Decrease ≥1 mm of joint space | Average 8.4 years |
| Birn 2014[[8](#_ENREF_8)] | Case-control | Secondary care, Radiology (USA) | Patients with hip complaints undergoing hip injection, diagnosis of OA or rapidly destructive OA | 94 (5 cases, 89 controls) | NR | 69 | 2006-2008 | Rapidly destructive OA: > 2 mm, or > 50% JSN/year | NR |
| Birrell 2003[[9](#_ENREF_9)] | Prospective cohort | Primary care (UK) | Patients with new episode of hip pain, arising from hip joint according to GP | 195 | 63±11 | 68 | 1994-1997 | Time to being put on waiting list for THR | 36 months |
| Bouyer 2016[[10](#_ENREF_10)] | Prospective cohort (KHOALA) | Population-based (FRA) | Participants, ACR criteria, 40-75 years, K-L grade ≥2 | 242 (analyzed 133 patients) | Median 62 inter-quartile range 57-48 | 71 | 2007-2009 | 1. Increase ≥1 K-L grade  2. Increase ≥1 JSN score  3. Time to THR | 3 years |
| Castano Betancourt 2009[[11](#_ENREF_11)] | Prospective cohort (GOAL) | Primary care (NED) | Patients, Clinical ACR criteria, no K-L grade 4, bilateral radiographs, DXA scan of adequate quality at baseline and 2 years follow-up | 189 | 63.5±9 | 69 | 2003- 2004 | JSN ≥ 20% compared to baseline or THR. | 2 years |
| Chaganti 2008[[12](#_ENREF_12)] | Nested case-control  (SOF) | Population-based (USA) | Participants, white ambulatory women, at least 65 years old at baseline, without bilateral hip replacement, with radiographic hip OA at baseline | 168 cases and 173 controls | Cases:  72.2 ±5.5  Controls: 71.8 ±5.5 | 100 | 1986-1988 | Decrease in MJS of 0.5 mm, or increase of ≥1 in summary grade, or increase ≥2 in total osteophyte score, or THR for OA | Average 8.3 years |
| Chevalier 2001[[13](#_ENREF_13)] | Prospective cohort | Secondary care, Rheumatology (FRA) | Patients, ACR criteria, K-L grade 2 or 3, VAS >40 mm | 30 | Females: 62±10  Males 55±13 | 55 | NR | Rapid evolution: JSN >0,6 mm/year | 1 year |
| Conrozier 1998_1[[14](#_ENREF_14)] | Case-control | NR | Symptomatic hip OA | 104 (analyzed 10 cases, 23 controls) | 63.7±12.33 | 61 | NR | Rapidly progressive hip OA: severe hip pain, symptom onset within the last 2 years, annual rate of JSN > 1mm, ESR < 20mm/h, absence of detectable inflammatory or crystal-induced joint disease | NR |
| Conrozier 1998_2[[15](#_ENREF_15)] | Retrospective cohort | Secondary care, Orthopedic Surgery (FRA) | Patients who had THR for OA. OA was defined as radiographic JSN and evidence of OA lesions of the femoral head and acetabulum at operation | 89 | 61.8±10.4 | 56 | 1992- 1993 | Radiographic: YMN, calculated from MJS in mm/year | 18-300 months |
| Conrozier 1998_3[[16](#_ENREF_16)] | Prospective cohort | Secondary care, Rheumatology (FRA) | Patients, symptomatic hip OA, ACR criteria, superior femoral head migration, | 48 | 56.4±14.1 | 52 | NR | JSN in mm/year | 1 year |
| Danielson 1964/ 1993 [[17](#_ENREF_17), [18](#_ENREF_18)] | Prospective cohort | Secondary care, Orthopedics (SWE) | Patients with hip complaints, radiographically primary coxarthrosis | 168 | 70.6±0.84 | 52 | 1950-1954 | 1. Increase in pain index 0-5  2. Operation because of hip OA  3. Increase in radiographic index 0-10 | 8-12 years |
| van Dijk 2010[[19](#_ENREF_19)] | Prospective cohort | Secondary care, Orthopedics, Rheumatology, Rehabilitation (NED) | Patients with radiologic of clinical ACR criteria, age 50-84 years, referral to a hospital or rehabilitation center < 1 year, Lequesne Algofunctional Index score ≥5 | 123 | 66.3±8.9 | 71 | NR | 1. decrease in WOMAC function  2. Increase in seconds of Timed walking test | 3 years |
| van Dijk 2011[[20](#_ENREF_20)] | Prospective cohort | Secondary care, Orthopedics, Rheumatology, Rehabilitation (NED) | Patients, Radiologic of clinical ACR criteria, age 50-84 years, referral to a hospital or rehabilitation center < 1 year, Lequesne Algofunctional Index score ≥5 | 123 | 66.3±8.9 | 71 | NR | 1. decrease in WOMAC function  2. Increase in seconds of Timed walking test | 3 years |
| Dorleijn 2015[[21](#_ENREF_21)] | Prospective cohort  (GOAL) | Primary care (NED) | Patients, clinical ACR criteria, no THR or not on the waiting list, no K-L grade 4, bilateral radiographs, DXA scan of adequate quality at baseline and 2 years follow-up | 222 (analyzed 111 patients) | 63.4±9 | 69 | 2003- 2004 | VAS score for pain, group highly progressive compared to mild pain Groups based on LCGA | 2 years |
| Dougados 1996[[22](#_ENREF_22)] | Prospective cohort  (ECHODIAH) | Secondary care, Rheumatology (FRA) | Patients, ACR criteria, 50-75 years, daily hip pain for ≥ 1 months during the past 2 months and a Lequesne algofunctional index of ≥3 points, JSW 1-3 mm | 508 (analyzed 461 patients) | 63±7 | 60 | NR | Radiological: ≥ 0.6 mm  decrease in JSW | 1 year |
| Dougados 1997[[23](#_ENREF_23)] | Prospective cohort (ECHODIAH) | Secondary care, Rheumatology (FRA) | Patients, ACR criteria, 50-75 years, daily hip pain for ≥ 1 months during the past 2 months and a Lequesne algofunctional index of ≥3 points, JSW 1-3 mm or if >3 mm than ≥ 0.5 mm thinner than contralateral hip | 508 (analyzed 463 patients) | 63±7 | 60 | NR | Radiological: >0,5 mm decrease in JSW | 2 years |
| Dougados 1999[[24](#_ENREF_24)] | Prospective cohort | Secondary care, Rheumatology (FRA) | Patients, ACR criteria, 50-75 years, daily pain ≥1 month in last 3 months, JSW > 1mm | 508 | NR | 60 | -NR | Time to requirement of THR | 3 years |
| Fukushima 2018[[25](#_ENREF_25)] | Prospective cohort | Secondary care, Orthopedics, Rehabilitation (JPN) |  | 20 | 42,9±3.0 | 65 | NR | Increase in Tönnis grade | 25 months |
| Golightly 2010[[26](#_ENREF_26)] | Prospective cohort (Johnston County) | Population-based (USA) | Participants, probability sampling, men ≥45 years, women >50 years, K-L grade ≥1 | 1453 | 61.9±9.6 | 61 | 1991-1997 | Increase in K- L grade or increase in hip symptoms (mild, moderate, severe) | 3-13 years |
| Gossec 2005[[27](#_ENREF_27)] | Prospective cohort | Secondary Care, Rheumatology (FRA) | Patients, ACR criteria, ≥40 years, hip pain > 6 months, pain VAS ≥ 30, pain ≥ 14 days during previous month | 741 (analyzed 505 patients) | 64.0±10.1 | 61 | NR | THR | 2 years |
| Hartofilakidis 2003[[28](#_ENREF_28)] | Retrospective cohort | Secondary care, Orthopedics (GRE) | Patients with hip problems, age of symptom onset > 45-50 years, normal laboratory tests | 210 | 61 SD: NR | 85 | 1970-1996 | THR | 2->10 years |
| Hawker 2006[[29](#_ENREF_29)] | Prospective cohort | Population-based (CAN) | Participants with self-reported difficulty in last 3 months with stair climbing, arising from chair, standing and walking, swelling, pain or stiffness in any joint ≥ 6 weeks, indication of troublesome hip and/or knee on diagram, WOMAC summary score ≥ 39 | 2128 | 71.5±9.5 | 73 | 1995-1997 | Time to THR | 6.1 years |
| Hoeven 2013[[30](#_ENREF_30)] | Prospective cohort  (Rotterdam I) | Population-based (NED) | Participants ≥ 55 years, radiographs of hips available and scored at baseline and follow-up | 5650 (number analyzed: NR) | 68.2±8 | 58 | 1989 - 1993 | Increase ≥ 1 K-L grade baseline to follow-up | 10 years |
| Holla 2010[[31](#_ENREF_31)] | Prospective cohort  (CHECK) | Combination of primary, secondary care and population-based (NED) | Patients and participants, 45-65 years, pain and/or stiffness in 1 or both knees and/or hips | 588 | 55.8±5.3 | 81 | 2002-2005 | Moving into higher group (quintiles of WOMAC-PF 0-68) or remaining within the 3 highest groups | 2 years |
| Juhakoski 2013[[32](#_ENREF_32)] | Prospective cohort | Population-based (FIN) | Participants, 55-80 years, K-L grade ≥1, pain in groin or lateral hip region | 118 | 66.6±6.4 | 70 | 2005-2006 | 1. WOMAC pain (0-100), 2. WOMAC function (0-100) | 2 years |
| Kalyoncu 2009[[33](#_ENREF_33)] | Retrolective cohort  (ECHODIAH) | Secondary care, Rheumatology (FRA) | Patients, ACR criteria, 50-75 years, daily hip pain for ≥ 1 months during the past 2 months and a Lequesne algofunctional index of ≥3 points, JSW 1-3 mm or if >3 mm than ≥ 0.5 mm thinner than contralateral hip | 192 | With psoriasis: 71.5±7.2 Without psoriasis: 71.8±6.2 | 59 | NR | THR | 10 year |
| Kelman 2006[[34](#_ENREF_34)] | Nested case-control  (SOF) | Population-based (USA) | Participants, Caucasian women, ≥65 years, baseline radio-graphic findings of hip OA: (1) a summary grade ≥2, (2) JSN severity grade ≥2 supero-laterally or severity grade ≥ 3 superomedially, or (3) definite osteophytes grade ≥2 in any location and definite JSN grade ≥2 in any location. | 396 (Cases 197, Controls 199) | Cases: 72.3±5.4  Controls: 71.9±5.8 | 100 | 1986 - 1988 | Decrease in minimum joint space of ≥ 0.5mm, or an increase of ≥1 in the summary grade, or an increase of ≥2 in total osteophyte score, or THR | 8.3 years |
| Kerkhof 2010[[35](#_ENREF_35)] | Prospective cohort  (Rotterdam I) | Population-based (NED) | Participants ≥ 55 years, K-L grade ≥1 | 1610 | Case*s:* 67.0±6.8 Controls: 65.4±6.6 | Cases: 61 Controls: 50 | 1989 - 1993 | Radiologic: JSN ≤1.0 mm or THR during follow-up | NR |
| Kopec 2013[[36](#_ENREF_36)] | Prospective cohort  (Johnston County) | Population-based (USA) | Participants, probability sampling, men ≥45 years, women >50 years, K-L grade 2-3 | 1590 (analyzed 571 people) | NR | 65 | 1991-1997 | Increase ≥1 in K-L grade | 3-13 years |
| Lane 2004[[37](#_ENREF_37)] | Prospective cohort  (SOF) | Population-based (USA) | Participants, Caucasian women, ≥65 years, baseline radiographic findings of hip OA: (1) a summary grade ≥2, (2) JSN severity grade ≥2 superolaterally or severity grade ≥ 3 superomedially, or (3) definite osteophytes grade ≥2 in any location and definite JSN grade ≥2 in any location | 745 | 71.8±5.2 | 100 | 1986 - 1988 | Decrease in minimum joint space of ≥ 0.5mm, or an increase of ≥1 in the summary grade, or an increase of ≥2 in total osteophyte score, or THR | 8 years |
| Lane 2007[[38](#_ENREF_38)] | Nested case-control  (SOF) | Population-based (USA) | Participants, Caucasian women, ≥65 years, baseline radiographic findings of hip OA: (1) a summary grade ≥2, (2) JSN severity grade ≥2 superolaterally or severity grade ≥ 3 superomedially, or (3) definite osteophytes grade ≥2 in any location and definite JSN grade ≥2 in any location | 342 | Cases: 72.4 ±5.6  Controls: 71.7±5.6 | 100 | 1986 -1988 | Radiological: decrease in minimum joint space of ≥ 0.5mm, or an increase of ≥1 in the summary grade, or an increase of ≥2 in total osteophyte score, or THR | 8.3 years |
| Laslett 2014[[39](#_ENREF_39)] | Prospective cohort  (TasOAC) | Population-based (Tasmania) | Participants 50-80 years, not living in aged-care facility, no contraindications for MRI | 1099 (analyzed 765 people) | 62.1±7 | 51 | 2002- 2004 | WOMAC pain (0-100) | 2,4 years |
| Ledingham 1993[[40](#_ENREF_40)] | Prospective cohort | Secondary care, Rheumatology and Orthopedics (UK) | Patients, hip OA: JSN with osteophytes, pain or clinical abnormalities at the hip | 136 | 65 SD: NR | 63 | NR | 1. Global assessment of radiographic change  2. THR | 3-73 months |
| Lievense 2007[[41](#_ENREF_41)] | Prospective cohort | Primary care (NED) | Patients ≥50 years, hip pain persisting for 1 month-2 years, GP requested a radiograph at participating hospital | 224  (analyzed 163 patients) | 65.6±9.6 | 73 | 1996 | THR | 5.8 years |
| Maillefert 2003[[42](#_ENREF_42)] | Prospective cohort  (ECHODIAH) | Secondary care, Rheumatology (FRA) | Patients, ACR criteria, 50-75 years, daily hip pain for ≥ 1 months during the past 2 months and a Lequesne algofunctional index of ≥3 points, JSW 1-3 mm or if >3 mm than ≥ 0.5 mm thinner than contralateral hip | 508 | 63±7 | 59 | NR | 1. Decrease in JSW >50% during first year follow-up 2. THR in 1 year -5 year of follow-up | 5 years |
| Mazieres 2006[[43](#_ENREF_43)] | Prospective cohort (ECHODIAH) | Secondary care, Rheumatology (FRA) | Patients, ACR criteria, 50-75 years, daily hip pain for ≥ 1 months during the past 2 months and a Lequesne algofunctional index of ≥3 points, JSW 1-3 mm or if >3 mm than ≥ 0.5 mm thinner than contralateral hip | 507 (analyzed 333 patients) | 62.1±6.9 | 59 | NR | JSN ≥0,5 mm or THP | 3 years |
| Nelson 2010[[44](#_ENREF_44)] | Prospective cohort  (Johnston County) | Population-based (USA) | Participants, probability sampling, men ≥45 years, women >50 years | 309 | 62.3±9.3 | 59 | 1991-1997 | 1. Increase in K-L grade. 2. Increase in osteophyte severity grade. 3. Increase in JSN severity grade | 5 years |
| Perry 1972[[45](#_ENREF_45)] | Case-control | Secondary care (UK) | Patients, normal erythrocyte sedimentation rate, a negative latex test, K-L grade ≥2, radiological cystic changes | 44 | NR | 78 | 1961 | Radiographic: progressive deterioration | 5-14 years |
| Peters 2005[[46](#_ENREF_46)] | Prospective cohort | Population-based in 40 general practices (UK) | Participants with hip pain | 587 (analyzed 214) | NR | NR | 1992-1994 | New Zealand score 0-80 (combination of pain and function) | 7 years |
| Pisters 2012[[47](#_ENREF_47)] | Prospective cohort | Secondary care, Orthopedics, Rheumatology and Rehabilitation (NED) | Patients, hip OA according to radiological or clinical ACR criteria, age 50-84 years, referral to hospital or rehabilitation center <1 year before inclusion, Lequesne Algofunctional index ≥ 5 | 149 | 66.7±9 | 71 | NR | Increase in WOMAC function on average over time (measured at 1,2,3, 5 years) | 5 years |
| Pollard 2012[[48](#_ENREF_48)] | Prospective cohort | Secondary care, 49 families with ≥ 2 female siblings undergone THA (UK) | Offspring (Sibkids) and their spouses (Controls) of family with ≥ 2 female siblings undergone THR | 264 | Sibkids: 52.3 ±8.1  Controls: 54.1±9.0 | Sibkids 50 Controls 51 | NR | Signs on examination of hip OA ór symptoms at baseline and signs ànd symptoms at follow-up | 5 years |
| Reijman 2004[[49](#_ENREF_49)] | Prospective cohort  (Rotterdam I) | Population-based (NED) | Participants ≥ 55 years, urine samples and radiographs of hips available and scored at baseline and follow-up. | 1235 | 66.6±6.8 | 58 | 1989 -1993 | JSN ≥1.0 mm in at least 1 of 3 compartments (lateral, superior, axial) | 6.6 years |
| Reijman 2005[[50](#_ENREF_50)] | Prospective cohort  (Rotterdam I) | Population-based (NED) | Participants ≥ 55 years, K-L grade ≥1 | 1904 | 66.2±7 | 53 | 1989 -1993 | Radiologic: JSN ≤1.0 mm or THR during follow-up | 6,6 years |
| Reijman 2007[[51](#_ENREF_51)] | Prospective cohort (Rotterdam I) | Population-based (NED) | Participants ≥ 55 years, K-L grade 1,2 or 3 | 1676 | 66.1±6.9 | 52 | 1989 - 1993 | 1. JSN of ≥1 mm. 2. JSN of ≥1.5 mm. 3. Increase of ≥1 K-L grade | 6,6 years |
| Solignac 2004[[52](#_ENREF_52)] | Prospective cohort  (ECHODIAH) | Secondary care, Rheumatology (FRA) | Patients complying with ACR criteria, 50-75 years, daily hip pain for ≥ 1 months during the past 2 months and a Lequesne algofunctional index of ≥3 points, JSW 1-3 mm | 507 (analyzed 333 patients) | 62.1±6.9 | 59 | NR | JSN ≥0,5 mm or THP | 3 years |
| van Spil 2015[[53](#_ENREF_53)] | Prospective cohort  (CHECK) | Combination of primary, secondary care and population-based (NED) | Patients and participants, 45-65 years, pain and/or stiffness in 1 or both knees and/or hips, K-L grade =1 | 1002  (analyzed 178 patients with hip pain) | 56 ±5 | 79 | 2002-2005 | Radiographic: ≥ 1 K-L grade increase | 5 years |
| Thompson 2004[[54](#_ENREF_54)] | Case-control | Secondary care (NI) | Patients undergone primary THA, no other disease entity | 34 cases, controls: NR | 72 SD: NR | 85 | 1991- 2001 | Rapidly progressive OA: loss of bone or a combined loss of bone and articular cartilage at rate >5mm per year | 18 months |
| Tron 1994[[55](#_ENREF_55)] | Retrospective cohort | Secondary care, Rheumatology (FRA) | Patients with symptomatic coxarthrosis | 39 | 55.5±12 | 54 | NR | Mean annual JSN in mm | NR |
| Verkleij 2012[[56](#_ENREF_56)] | Prospective cohort (GOAL) | Primary care (NED) | Patients, ACR criteria, no K-L grade 4. | 222 (analyzed 111 patients) | 63.4±9 | 69 | 2003-2004 | VAS score for pain, group highly progressive compared to mild pain  Groups based on LCGA | 2 years |
| Vinciguerra 1995[[57](#_ENREF_57)] | Retrospective cohort | Secondary care, Rheumatology (FRA) | Patients, ACR criteria | 149 | 58±14 | 66 | 1981-1986 | Time to THR | Variable |

Study population: the most important criteria are presented.
Abbreviations: n=number. SD=standard deviation. NR=not reported. OA=osteoarthritis. THR=total hip replacement. K-L grade=Kellgren and Lawrence grade. ACR=American College of Rheumatology. NED=the Netherlands. JPN=Japan. UK=United Kingdom. USA=United States of America. FRA=France. SWE=Sweden. CAN=Canada. NI= North Ireland. GRE=Greece. FIN=Finland. MJS=minimum joint space. JSN=joint space narrowing. JSW=joint space width. YMN=yearly mean narrowing. LCGA=latent class growth analysis. ESR=erythrocyte sedimentation rate. NRS=numeric rating scale. VAS=visual analogue scale.

References

1. Agricola R, Reijman M, Bierma-Zeinstra SMA, Verhaar JAN, Weinans H, Waarsing JH: **Total hip replacement but not clinical osteoarthritis can be predicted by the shape of the hip: A prospective cohort study (CHECK)**. *Osteoarthr Cartilage* 2013, **21**(4):559-564.

2. Agricola R, Leyland KM, Bierma-Zeinstra SM, Thomas GE, Emans PJ, Spector TD, Weinans H, Waarsing JH, Arden NK: **Validation of statistical shape modelling to predict hip osteoarthritis in females: data from two prospective cohort studies (Cohort Hip and Cohort Knee and Chingford)**. *Rheumatology (Oxford)* 2015, **54**(11):2033-2041.

3. Auquier L, Paolaggi JB, Cohen De Lara A: **Long term evolution of pain in a series of 273 coxarthrosis**. *Rev Rhum Mal Osteo-Articul* 1979, **46**(3):153-162.

4. Barr RJ, Gregory JS, Reid DM, Aspden RM, Yoshida K, Hosie G, Silman AJ, Alesci S, Macfarlane GJ: **Predicting OA progression to total hip replacement: Can we do better than risk factors alone using active shape modelling as an imaging biomarker?** *Rheumatology (Oxford)* 2012, **51**(3):562-570.

5. Bastick AN, Verkleij SPJ, Damen J, Wesseling J, Hilberdink WKHA, Bindels PJE, Bierma-Zeinstra SMA: **Defining hip pain trajectories in early symptomatic hip osteoarthritis - 5 year results from a nationwide prospective cohort study (CHECK)**. *Osteoarthritis Cartilage* 2016, **24**(5):768-775.

6. Bastick AN, Damen J, Agricola R, Brouwer RW, Bindels PJ, Bierma-Zeinstra SM: **Characteristics associated with joint replacement in early symptomatic knee or hip osteoarthritis: 6-year results from a nationwide prospective cohort study (CHECK)**. *Br J Gen Pract* 2017, **67**(663):e724-e731.

7. Bergink AP, Zillikens MC, Van Leeuwen JPTM, Hofman A, Uitterlinden AG, van Meurs JBJ: **25-Hydroxyvitamin D and osteoarthritis: A meta-analysis including new data**. *Semin Arthritis Rheum* 2016, **45**(5):539-546.

8. Birn J, Pruente R, Avram R, Eyler W, Mahan M, van Holsbeeck M: **Sonographic evaluation of hip joint effusion in osteoarthritis with correlation to radiographic findings**. *J Clin Ultrasound* 2014, **42**(4):205-211.

9. Birrell F, Afzal C, Nahit E, Lunt M, Macfarlane GJ, Cooper C, Croft PR, Hosie G, Silman AJ: **Predictors of hip joint replacement in new attenders in primary care with hip pain**. *Br J Gen Pract* 2003, **53**(486):26-30.

10. Bouyer B, Mazieres B, Guillemin F, Bouttier R, Fautrel B, Morvan J, Pouchot J, Rat AC, Roux CH, Verrouil E *et al*: **Association between hip morphology and prevalence, clinical severity and progression of hip osteoarthritis over 3 years: The knee and hip osteoarthritis long-term assessment cohort results**. *Jt Bone Spine* 2016, **83**(4):432-438.

11. Castano Betancourt MC, Van der Linden JC, Rivadeneira F, Rozendaal RM, Bierma Zeinstra SM, Weinans H, Waarsing JH: **Dual energy x-ray absorptiometry analysis contributes to the prediction of hip osteoarthritis progression**. *Arthritis Res Ther* 2009, **11**(6):R162.

12. Chaganti RK, Kelman A, Lui L, Yao W, Javaid MK, Bauer D, Nevitt M, Lane NE, for the Study of Osteoporotic Fractures Research G: **Change in serum measurements of cartilage oligomeric matrix protein and association with the development and worsening of radiographic hip osteoarthritis**. *Osteoarthr Cartilage* 2008, **16**(5):566-571.

13. Chevalier X, Conrozier T, Gehrmann M, Claudepierre P, Mathieu P, Unger S, Vignon E: **Tissue inhibitor of metalloprotease-1 (TIMP-1) serum level may predict progression of hip osteoarthritis**. *Osteoarthr Cartilage* 2001, **9**(4):300-307.

14. Conrozier T, Chappuis-Cellier C, Richard M, Mathieu P, Richard S, Vignon E: **Increased serum C-reactive protein levels by immunonephelometry in patients with rapidly destructive hip osteoarthritis**. *Rev Rhum Engl Ed* 1998, **65**(12):759-765.

15. Conrozier T, Jousseaume CA, Mathieu P, Tron AM, Caton J, Bejui J, Vignon E: **Quantitative measurement of joint space narrowing progression in hip osteoarthritis: A longitudinal retrospective study of patients treated by total hip arthroplasty**. *Br J Rheumatol* 1998, **37**(9):961-968.

16. Conrozier T, Saxne T, Fan CSS, Mathieu P, Tron AM, Heinegard D, Vignon E: **Serum concentrations of cartilage oligomeric matrix protein and bone sialoprotein in hip osteoarthritis: A one year prospective study**. *Ann Rheum Dis* 1998, **57**(9):527-532.

17. Danielsson LG: **Incidence and prognosis of coxarthrosis**. *Acta Orthop Scand Suppl* 1964, **66**:SUPPL 66:61-114.

18. Danielsson LG: **Incidence and prognosis of coxarthrosis**. *Clin Orthop Relat Res* 1993(287):13-18.

19. van Dijk GM, Veenhof C, Spreeuwenberg P, Coene N, Burger BJ, van Schaardenburg D, van den Ende CH, Lankhorst GJ, Dekker J: **Prognosis of Limitations in Activities in Osteoarthritis of the Hip or Knee: A 3-Year Cohort Study**. *Arch Phys Med Rehabil* 2010, **91**(1):58-66.

20. Van Dijk GM, Veenhof C, Lankhorst GJ, Van Den Ende CH, Dekker J: **Vitality and the course of limitations in activities in osteoarthritis of the hip or knee**. *BMC Musculoskelet Disord* 2011, **12**:269.

21. Dorleijn DMJ, Luijsterburg PAJ, Bay-Jensen AC, Siebuhr AS, Karsdal MA, Rozendaal RM, Bos PK, Bierma-Zeinstra SMA: **Association between biochemical cartilage markers and clinical symptoms in patients with hip osteoarthritis: Cohort study with 2-year follow-up**. *Osteoarthr Cartilage* 2015, **23**(1):57-62.

22. Dougados M, Gueguen A, Nguyen M, Berdah L, Lequesne M, Mazieres B, Vignon E: **Radiological progression of hip osteoarthritis: Definition, risk factors and correlations with clinical status**. *Ann Rheum Dis* 1996, **55**(6):356-362.

23. Dougados M, Gueguen A, Nguyen M, Berdah L, Lequesne M, Mazieres B, Vignon E: **Radiographic features predictive of radiographic progression of hip osteoarthritis**. *Rev Rhum Engl Ed* 1997, **64**(12):795-803.

24. Dougados M, Gueguen A, Nguyen M, Berdah L, Lequesne M, Mazieres B, Vignon E: **Requirement for total hip arthroplasty: An outcome measure of hip osteoarthritis?** *J Rheumatol* 1999, **26**(4):855-861.

25. Fukushima K, Inoue G, Uchida K, Fujimaki H, Miyagi M, Nagura N, Uchiyama K, Takahira N, Takaso M: **Relationship between synovial inflammatory cytokines and progression of osteoarthritis after hip arthroscopy: Experimental assessment**. *J Orthop Surg* 2018, **26**(2).

26. Golightly YM, Allen KD, Helmick CG, Schwartz TA, Renner JB, Jordan JM: **Hazard of incident and progressive knee and hip radiographic osteoarthritis and chronic joint symptoms in individuals with and without limb length inequality**. *J Rheumatol* 2010, **37**(10):2133-2140.

27. Gossec L, Tubach F, Baron G, Ravaud P, Logeart I, Dougados M: **Predictive factors of total hip replacement due to primary osteoarthritis: A prospective 2 year study of 505 patients**. *Ann Rheum Dis* 2005, **64**(7):1028-1032.

28. Hartofilakidis G, Karachalios T: **Idiopathic osteoarthritis of the hip: Incidence, classification, and natural history of 272 cases**. *Orthopedics* 2003, **26**(2):161-166.

29. Hawker GA, Guan J, Croxford R, Coyte PC, Glazier RH, Harvey BJ, Wright JG, Williams JI, Badley EM: **A prospective population-based study of the predictors of undergoing total joint arthroplasty**. *Arthritis Rheum* 2006, **54**(10):3212-3220.

30. Hoeven TA, Kavousi M, Clockaerts S, Kerkhof HJM, Van Meurs JB, Franco O, Hofman A, Bindels P, Witteman J, Bierma-Zeinstra S: **Association of atherosclerosis with presence and progression of osteoarthritis: The Rotterdam Study**. *Ann Rheum Dis* 2013, **72**(5):646-651.

31. Holla JFM, Steultjens MPM, Roorda LD, Heymans MW, Ten Wolde S, Dekker J: **Prognostic factors for the two-year course of activity limitations in early osteoarthritis of the hip and/or knee**. *Arthritis Care Res* 2010, **62**(10):1415-1425.

32. Juhakoski R, Malmivaara A, Lakka TA, Tenhonen S, Hannila ML, Arokoski JP: **Determinants of pain and functioning in hip osteoarthritis - a two-year prospective study**. *Clin Rehabil* 2013, **27**(3):281-287.

33. Kalyoncu U, Gossec L, Nguyen M, Berdah L, Mazieres B, Lequesne M, Dougados M: **Self-reported prevalence of psoriasis and evaluation of the impact on the natural history of hip osteoarthritis: Results of a 10 years follow-up study of 507 patients (ECHODIAH study)**. *Jt Bone Spine* 2009, **76**(4):389-393.

34. Kelman A, Lui L, Yao W, Krumme A, Nevitt M, Lane NE: **Association of higher levels of serum cartilage oligomeric matrix protein and N-telopeptide crosslinks with the development of radiographic hip osteoarthritis in elderly women**. *Arthritis Rheum* 2006, **54**(1):236-243.

35. Kerkhof HJM, Bierma-Zeinstra SMA, Castano-Betancourt MC, De Maat MP, Hofman A, Pols HAP, Rivadeneira F, Witteman JC, Uitterlinden AG, Van Meurs JBJ: **Serum C reactive protein levels and genetic variation in the CRP gene are not associated with the prevalence, incidence or progression of osteoarthritis independent of body mass index**. *Ann Rheum Dis* 2010, **69**(11):1976-1982.

36. Kopec JA, Sayre EC, Schwartz TA, Renner JB, Helmick CG, Badley EM, Cibere J, Callahan LF, Jordan JM: **Occurrence of radiographic osteoarthritis of the knee and hip among african americans and whites: A population-based prospective cohort study**. *Arthritis Care Res* 2013, **65**(6):928-935.

37. Lane NE, Nevitt MC, Hochberg MC, Hung YY, Palermo L: **Progression of Radiographic Hip Osteoarthritis over Eight Years in a Community Sample of Elderly White Women**. *Arthritis Rheum* 2004, **50**(5):1477-1486.

38. Lane NE, Nevitt MC, Lui LY, De Leon P, Corr M: **Wnt signaling antagonists are potential prognostic biomarkers for the progression of radiographic hip osteoarthritis in elderly Caucasian women**. *Arthritis Rheum* 2007, **56**(10):3319-3325.

39. Laslett LL, Quinn S, Burgess JR, Parameswaran V, Winzenberg TM, Jones G, Ding C: **Moderate vitamin D deficiency is associated with changes in knee and hip pain in older adults: a 5-year longitudinal study**. *Ann Rheum Dis* 2014, **73**(4):697-703.

40. Ledingham J, Dawson S, Preston B, Milligan G, Doherty M: **Radiographic progression of hospital referred osteoarthritis of the hip**. *Ann Rheum Dis* 1993, **52**(4):263-267.

41. Lievense AM, Koes BW, Verhaar JAN, Bohnen AM, Bierma-Zeinstra SMA: **Prognosis of hip pain in general practice: A prospective followup study**. *Arthritis Care Res* 2007, **57**(8):1368-1374.

42. Maillefert JF, Gueguen A, Monreal M, Nguyen M, Berdah L, Lequesne M, Mazieres B, Vignon E, Dougados M: **Sex differences in hip osteoarthritis: Results of a longitudinal study in 508 patients**. *Ann Rheum Dis* 2003, **62**(10):931-934.

43. Mazieres B, Garnero P, Gueguen A, Abbal M, Berdah L, Lequesne M, Nguyen M, Salles JP, Vignon E, Dougados M: **Molecular markers of cartilage breakdown and synovitis at baseline as predictors of structural progression of hip osteoarthritis. The ECHODIAH* cohort**. *Ann Rheum Dis* 2006, **65**(3):354-359.

44. Nelson AE, Golightly YM, Kraus VB, Stabler T, Renner JB, Helmick CG, Jordan JM: **Serum transforming growth factor-beta 1 is not a robust biomarker of incident and progressive radiographic osteoarthritis at the hip and knee: The Johnston County Osteoarthritis Project**. *Osteoarthr Cartilage* 2010, **18**(6):825-829.

45. Perry GH, Smith MJ, Whiteside CG: **Spontaneous recovery of the joint space in degenerative hip disease**. *Ann Rheum Dis* 1972, **31**(6):440-448.

46. Peters TJ, Sanders C, Dieppe P, Donovan J: **Factors associated with change in pain and disability over time: A community-based prospective observational study of hip and knee osteoarthritis**. *Br J Gen Pract* 2005, **55**(512):205-211.

47. Pisters MF, Veenhof C, van Dijk GM, Heymans MW, Twisk JWR, Dekker J: **The course of limitations in activities over 5 years in patients with knee and hip osteoarthritis with moderate functional limitations: Risk factors for future functional decline**. *Osteoarthr Cartilage* 2012, **20**(6):503-510.

48. Pollard TCB, Batra RN, Judge A, Watkins B, McNally EG, Gill HS, Arden NK, Carr AJ: **Genetic predisposition to the presence and 5-year clinical progression of hip osteoarthritis**. *Osteoarthr Cartilage* 2012, **20**(5):368-375.

49. Reijman M, Hazes JMW, Bierma-Zeinstra SMA, Koes BW, Christgau S, Christiansen C, Uitterlinden AG, Pols HAP: **A new marker for osteoarthritis: Cross-sectional and longitudinal approach**. *Arthritis Rheum* 2004, **50**(8):2471-2478.

50. Reijman M, Hazes JMW, Pols H, Bernsen RMD, Koes BW, Bierma-Zeinstra SMA: **Role of radiography in predicting progression of osteoarthritis of the hip: Prospective cohort study**. *Br Med J* 2005, **330**(7501):1183-1185.

51. Reijman M, Pols HAP, Bergink AP, Hazes JMW, Belo JN, Lievense AM, Bierma-Zeinstra SMA: **Body mass index associated with onset and progression of osteoarthritis of the knee but not of the hip: The Rotterdam Study**. *Ann Rheum Dis* 2007, **66**(2):158-162.

52. Solignac M: **Biological markers of osteoarthritis: data from the ECHODIAH cohort**. *Presse Med* 2004, **33**(9 Pt 2):S13-15.

53. Van Spil WE, Welsing PM, Bierma-Zeinstra SM, Bijlsma JW, Roorda LD, Cats HA, Lafeber FP: **The ability of systemic biochemical markers to reflect presence, incidence, and progression of early-stage radiographic knee and hip osteoarthritis: data from CHECK**. *Osteoarthr Cartilage* 2015, **23**(8):1388-1397.

54. Thompson NW, Corr AM, Geddis CJ, O'Brien S, Beverland DE: **Rapidly progressive osteoarthrosis of the hip**. *HIP Int* 2004, **14**(4):217-222.

55. Tron AM, Conrozier T, Mathieu P, Vignon E: **[Rate of joint space pinching in coxarthrosis] Vitesse de pincement de l'interligne articulaire dans la coxarthrose**. *Rev Rhum Ed Fr* 1994, **61**(9 Pt 2):124S-130S.

56. Verkleij SPJ, Hoekstra T, Rozendaal RM, Waarsing JH, Koes BW, Luijsterburg PAJ, Bierma-Zeinstra SMA: **Defining discriminative pain trajectories in hip osteoarthritis over a 2-year time period**. *Ann Rheum Dis* 2012, **71**(9):1517-1523.

57. Vinciguerra C, Gueguen A, Revel M, Heuleu JN, Amor B, Dougados M: **Predictors of the need for total hip replacement in patients with osteoarthritis of the hip**. *Rev Rhum Engl Ed* 1995, **62**(9):563-570.
