# Supplementary material for: Prognostic factors for progression of osteoarthritis of the hip: a systematic review
Source: Arthritis Res Ther. 2019 Aug 23;21:192. doi: 10.1186/s13075-019-1969-9 (PMC6708123; doi:10.1186/s13075-019-1969-9)
Supplement: Supplementary file 4 — Prognostic factors described by one study or multiple studies from the same cohort. (DOCX 126 kb) [file 13075_2019_1969_MOESM4_ESM.docx]

Additional file 4. Prognostic factors described by one study or multiple studies from the same cohort.

| Prognostic factor^ref^ | Results | Risk of bias assessment per domain of study | | | | | | | | | | |  |
| --- | --- | --- | --- | --- | --- | --- | --- | --- | --- | --- | --- | --- | --- |
|  |  | Study Participation | Study Attrition | | Prognostic Factor Measurement | | Outcome Measurement | | Study Confounding | | Statistical Analysis/ Reporting | |  |
| *Patient variables* | | | | | | | | | | | | |  |
| Age at menopause[[1](#_ENREF_1)] | Categorical groups of ≤45, 46-50 or >50 years. Progression was JSN ≤1.0 mm or THP during follow-up. Group ≤45 years compared to >50 years OR 1.3 (95% CI 0.9; 1.9). Group 46-50 years compared to >50 years OR 1.6 (95% CI 1.1; 1.6). | Low | Moderate | | Low | | Low | | Low | | Low | |  |
| Social class[[2](#_ENREF_2)] | Progression was increase in New Zealand score. In univariate analysis social class was not significantly associated with progression. | Low | Moderate | | Moderate | | Low | | Moderate | | Low | |  |
| Marital status[[2](#_ENREF_2)] | Categorical groups of partner, no partner or widowed. Progression was increase in New Zealand score. In univariate analysis marital status was not significantly associated with progression. | Low | Moderate | | Moderate | | Low | | Moderate | | Low | |  |
| Annual household income[[3](#_ENREF_3)] | Categorical groups of ≤$20,000, $20,000-$40,000 or >$40,000. Progression was time to THR. $20,000-$40,000 compared to ≤$20,000 HR 1.00 (95% CI 0.74; 1.36). >$40,000 compared to ≤$20,000 HR 0.61(95% CI 0.34; 1.10). | Moderate | Low | | Low | | Low | | Low | | Low | |  |
| Region[[3](#_ENREF_3)] | Categorical groups of Urban or Rural. Progression was time to THR. Urban compared to Rural HR 0.99 (95% CI 0.77; 1.27). | Moderate | Low | | Low | | Low | | Low | | Low | |  |
| Employment as farmer[[3](#_ENREF_3)] | Categorical groups of current farmer or non-farmer. Progression was time to THR. Farmer compared to non-farmer HR 1.16 (95%CI 0.77; 1.75). | Moderate | Low | | Low | | Low | | Low | | Low | |  |
| Employment status partner[[2](#_ENREF_2)] | Progression was increase in New Zealand score. In univariate analysis employment status of partner was not significantly associated with progression. | Low | Moderate | | Moderate | | Low | | Moderate | | Low | |  |
| *Disease characteristics* |  |  |  | |  | |  | |  | |  | |  |
| Limb length inequality[[4](#_ENREF_4)]  (≥2 cm difference in length between limbs) | Progression defined as increase ≥1 K-L grade if K-L grade was ≥1 at baseline: HR 1.32 (95% CI 0.78; 2.22). Progression defined as Increase ≥1 K-L grade if K-L grade was ≥2 at baseline: HR 1.34 (95% CI 0.37; 4.83). Progression defined as an increase in hip symptoms (pain, aching, or stiffness) from mild or moderate symptoms at baseline to moderate or severe symptoms at follow-up: HR 0.95 (95% CI 0.23-3.94). | Low | Moderate | | Low | | Low | | Low | | Low | |  |
| Concurrent OA of the hand[[1](#_ENREF_1)] | Definition of Hand OA: K-L grade ≥2 in ≥1-2 joints out of 2 groups (distal interphalangeal, proximal interphalangeal and first carpometacarpal). Progression was JSN ≤1.0 mm or THP during follow-up. OR 2.0 (95% CI 1.5; 2.6). | Low | Moderate | | Low | | Low | | Low | | Low | |  |
| Concurrent back pain[[5](#_ENREF_5)] | Progression based on pain trajectories from LCGA. Pain trajectory highly progressive compared to pain trajectory mild pain. OR 2.6 (95% CI 1.1; 5.9). | Low | Low | | Low | | Low | | Moderate | | Low | |  |
| Concurrent trochanteric pain[[5](#_ENREF_5)] | Progression based on pain trajectories from LCGA. Pain trajectory highly progressive compared to pain trajectory mild pain. OR 1.0 (95% CI 0.5; 2.2). | Low | Low | | Low | | Low | | Moderate | | Low | |  |
| Hypercholesterolemia[[6](#_ENREF_6)] | Definition was an increase of 30% compared to normal levels and/or treatment for disease. Progression was YMN, calculated from mean JSW in mm/year. In univariate analysis hypercholesterolemia was not significantly associated with progression. | Moderate | Low | | Low | | Low | | Low | | Low | |  |
| Cardiovascular comorbidity[[2](#_ENREF_2)] | Progression was increase in New Zealand score. In people with cardiovascular comorbidity New Zealand score was 7.1 (95% CI 1.6; 12.6) higher than in people without cardiovascular comorbidity. | Low | Moderate | | Moderate | | Low | | Moderate | | Low | |  |
| Atherosclerosis[[7](#_ENREF_7)] | Progression was increase ≥1 K-L grade. In men they found no association between atherosclerosis and progression. In women adjusted OR 0.5 (95% CI 0.17; 1.17) for 1 mm increase in carotid intima media thickness, and adjusted OR 0.7 (95% CI 0.53; 1.02) for presence of plaque in the carotid artery. | Low | Moderate | | Low | | Low | | Moderate | | Low | |  |
| Eye disease[[2](#_ENREF_2)] | Progression was increase in New Zealand score. In univariate analysis there was no significant association found. | Low | Moderate | | Moderate | | Low | | Moderate | | Low | |  |
| Other comorbidity[[2](#_ENREF_2)] (other than cardio-vascular, respiratory, eye diseases, hypertension, depression or cancer) | Progression was increase in New Zealand score. Patients with another comorbidity had 5.1 higher score (range 0-80) than patients without another comorbidity (95% CI 0.7; 9.6). | Low | Moderate | | Moderate | | Low | | Moderate | | Low | |  |
| Chondrocalcinosis[[8](#_ENREF_8)] | Chondrocalcinosis did not influence radiographic progression (global assessment of change by radiologist). Chondrocalcinosis showed no association with the need for THP. | Moderate | Moderate | | Moderate | | High | | High | | High | |  |
| Self-reported psoriasis[[9](#_ENREF_9)] | Progression was THR rate over 10 years. No significant association between psoriasis and progression was found. | Low | Low | | Moderate | | Moderate | | Low | | Low | |  |
| Cancer[[2](#_ENREF_2)] | Progression was increase in New Zealand score. In univariate analysis having cancer was not significantly associated with progression. | Low | Moderate | | Moderate | | Low | | Moderate | | Low | |  |
| Affected side[[10](#_ENREF_10)] | Progression was THR. In multivariate analysis, the number of THP at 6 years of follow-up did not differ between the right and left hip. | Low | Low | | Moderate | | Low | | Low | | Low | |  |
| Tenderness by palpation[[10](#_ENREF_10)] | Progression was THR. In univariate analysis; trochanteric tenderness OR 0.9 (p= 0.69), inguinal ligament tenderness OR 2.0 (p=0.07), superior iliac posterior spines tenderness OR 0.8 (p=0.47), sacroiliac joint tenderness OR 1.5 (p=0.26), ischial nerve tenderness OR 0.4 (p=0.20). Groin tenderness: OR 2.9 (p=0.01) in univariate analysis. No significant association was found in multivariate analysis for groin tenderness. | Low | Low | | Moderate | | Low | | Low | | Low | |  |
| Worst pain distribution[[10](#_ENREF_10)] | Progression was THR. In univariate analysis; trochanteric tenderness OR 0.7 (p= 0.35), anterior thigh OR 1.7 (p=0.37), lateral thigh OR <0.01 (p=0.68), buttock OR 0.3 (p=0.02). No significant association was found in multivariate analysis for buttock as worst pain distribution. In multivariate analysis: groin OR 2.8 (95% CI 1.0; 8.1) and medial thigh OR 14.1 (95% CI 0.4; 457.2). | Low | Low | | Moderate | | Low | | Low | | Low | |  |
| Pain at straight leg raising[[10](#_ENREF_10)] | Progression was THR. OR 0.2 (p=0.12) in univariate analysis. | Low | Low | | Moderate | | Low | | Low | | Low | |  |
| Painful passive hip motion in extension[[10](#_ENREF_10)] | Progression was THR. OR 1.4 (p=0.36) in univariate analysis. | Low | Low | | Moderate | | Low | | Low | | Low | |  |
| Pain aggravation[[10](#_ENREF_10)] | Progression was THR. In univariate analysis; pain aggravation by sitting OR 0.6 (p= 0.26), by only moving the hip joint OR 1.9 (p=0.08), by lying on the side OR 0.8 (p=0.54), by walking OR 2.4 (p=0.06), by after load OR 2.2 (p=0.04), on initial step after rest OR1.1 (p=0.80). No significant association was found in multivariate analysis for pain aggravation by lying only moving the hip joint, by walking or by after load. | Low | Low | | Moderate | | Low | | Low | | Low | |  |
| Decreased active hip motion with extension[[10](#_ENREF_10)] | Progression was THR. OR 2.2 (95% CI 0.8; 6.4) in multivariate analysis. | Low | Low | | Moderate | | Low | | Low | | Low | |  |
| Decreased active hip motion with adduction[[10](#_ENREF_10)] | Progression was THR. OR 2.1 (95% CI 0.8; 5.8) in multivariate analysis. | Low | Low | | Moderate | | Low | | Low | | Low | |  |
| Decreased active hip motion with abduction[[10](#_ENREF_10)] | Progression was THR. OR 3.6 (p=0.01) in univariate analysis. In multivariate analysis no significant association with progression was found. | Low | Low | | Moderate | | Low | | Low | | Low | |  |
| Trendelenburg’s sign[[10](#_ENREF_10)] | Progression was THR. A positive Trendelenburg’s sign was not significantly associated with progression in univariate analysis. | Low | Low | | Moderate | | Low | | Low | | Low | |  |
| Exercise tolerance[[8](#_ENREF_8)] | A decrease in exercise tolerance was associated with THR during follow-up, OR 2.68 (95%CI 1.29; 5.60). | Moderate | Moderate | | Moderate | | High | | High | | High | |  |
| Inflammatory arthritis type of hip OA[[3](#_ENREF_3)] | Progression was defined as time to THR. Inflammatory type compared to non-inflammatory type: HR 0.86 (95% CI 0.53; 1.39). | Moderate | Low | | Low | | Low | | Low | | Low | |  |
| *Chemical or Imaging Markers* | |  | |  | |  | |  | |  | |  | |
| Metalloproteinase derived type II collagen neoepitope[[11](#_ENREF_11)] (CIIM, measured in urine pg/umol creat) | Progression based on pain trajectories from LCGA. Pain trajectory highly progressive compared to pain trajectory mild pain. Increase of 1 unit in log pg/umol creat: OR 1.7 (95% CI 0.4; 7.2). | Low | Low | | Moderate | | Low | | Moderate | | Low | |  |
| Frizzled-related protein[[12](#_ENREF_12)] (FRP, measured in serum ng/ml) | Progression was defined as a decrease in minimum joint space of ≥ 0.5mm, or an increase of ≥1 in the summary grade, or an increase of ≥2 in total osteophyte score, or THR. Per 1 standard deviation increase in ng/ml: OR 0.95 (95% CI 0.75; 1.19). | Moderate | Low | | Moderate | | Low | | Low | | Low | |  |
| Tissue inhibitor metalloproteinases-1[[13](#_ENREF_13)] (TIMP-1, measured in serum ng/ml) | Progression was defined as rapid radiological evolution (JSN >0.6 mm/year). In patients with progression TIMP-1 was 173 ng/ml lower than in patients without progression (p=0.01). | Moderate | Low | | Low | | Low | | Moderate | | Moderate | |  |
| Dkk-1[[12](#_ENREF_12)] (measured in serum ng/ml) | Progression was defined as a decrease in minimum joint space of ≥ 0.5mm, or an increase of ≥1 in the summary grade, or an increase of ≥2 in total osteophyte score, or THR. Per 1 standard deviation increase in ng/ml: OR 0.75 (95% CI 0.52; 1.10).  For 1 unit increase in vitamin D, WOMAC pain score was 0.01 higher (95% CI -0.01; 0.04). | Moderate | Low | | Moderate | | Low | | Low | | Low | |  |
| Transforming growth factor-beta 1[[14](#_ENREF_14)]  (TGF-β1, measured in serum ng/ml) | Progression defined as an increase in K-L grade ≥1 if baseline K-L grade ≥1: HR 1.02 (95% CI 0.46;2.28) per 1 unit increase in log ng/ml. Progression defined as an increase K-L grade ≥1 if baseline K-L grade ≥2: HR 2.74 (95% CI 0.31;24.30) per 1 unit increase in log ng/ml. Progression defined as osteophyte severity grade increase ≥1 grade if baseline ≥1 grade: HR 0.55 (95% CI 0.12;2.59) per 1 unit increase in log ng/ml. Progression defined as JSN severity grade increase ≥1 grade if baseline ≥1 grade: HR 4.14 (95% CI 0.62;27.7) per 1 unit increase in log ng/ml. | Low | Moderate | | Low | | Low | | Low | | Low | |  |
| Bone sialoprotein[[15](#_ENREF_15)] (BSP, measured in serum ng/ml) | Progression was defined as YMN in mm/year. There was no significant association found between concentration of BSP and progression. | Moderate | Low | | Low | | Low | | Low | | Low | |  |
| Synovial tumour necrosis factor α (TNFα)[[16](#_ENREF_16)] | Progression was defined as progression in Tönnis grade. A higher cytokine level of synovial TNFα at baseline was found in the group of patients who showed progression (p=0.042) | Moderate | Low | | Low | | High | | High | | Low | |  |
| Synovial interleukin 1β (IL1β )[[16](#_ENREF_16)] | Progression was defined as progression in Tönnis grade. No significant difference in cytokine level of synovial IL1β at baseline was found between the patients with and without progression (p=0.67). | Moderate | Low | | Low | | High | | High | | Low | |  |
| Synovial interleukin 6  (IL6 )[[16](#_ENREF_16)] | Progression was defined as progression in Tönnis grade. No significant difference in cytokine level of synovial IL6 at baseline was found between the patients with and without progression (p=0.60). | Moderate | Low | | Low | | High | | High | | Low | |  |
| Synovial A disintegrin and metalloproteinase with thrombospondin motifs 4 (ADAMTS4)[[16](#_ENREF_16)] | Progression was defined as progression in Tönnis grade. No significant difference in cytokine level of synovial ADAMTS4 at baseline was found between the patients with and without progression (p=0.51). | Moderate | Low | | Low | | High | | High | | Low | |  |
| Synovial matrix metalloproteinase 1  (MMP-1)[[16](#_ENREF_16)] | Progression was defined as progression in Tönnis grade. No significant difference in cytokine level of synovial MMP-1 at baseline was found between the patients with and without progression (p=0.84). | Moderate | Low | | Low | | High | | High | | Low | |  |
| Synovial matrix metalloproteinase 3  (MMP-3)[[16](#_ENREF_16)] | Progression was defined as progression in Tönnis grade. No significant difference in cytokine level of synovial MMP-3 at baseline was found between the patients with and without progression (p=0.74). | Moderate | Low | | Low | | High | | High | | Low | |  |
| Croft grade at baseline[[17](#_ENREF_17)] | Progression defined as time to being put on the waiting for THR during 36 months of follow-up. Croft grades were compared, reference group was Croft grade 0 or 1. Croft grade 2; HR 3.36 (95% CI 0.31; 38.19), Croft grade 3; HR 15.23 (95% CI 3.29; 70.49), Croft grade 4; HR 44.51 (95% CI 10.04; 197.48) and Croft grade 5: HR 57.29 (95% CI 12.12; 270.71), p<0.0001. Hazard ratios were adjusted for age and gender. | Low | Low | | Moderate | | Low | | Low | | Low | |  |
| Tönnis grade at baseline[[16](#_ENREF_16)] | Progression was defined as progression in Tönnis grade. More patients with a high Tönnis grade 1 or 2 progressed to a higher Tönnis grade than patients with a Tönnis grade 0 at baseline (p=0.016). | Moderate | Low | | Low | | High | | High | | Low | |  |
| Radiographic findings of femoral acetabular impingement at baseline[[16](#_ENREF_16)] | Progression was defined as progression in Tönnis grade. 25% of patients with radiographic findings of femoral acetabular impingement at baseline showed progession, while 69% of patients without radiographic findings of femoral acetabular impingement showed progression, this difference was not statistical significant (p=0.26). | Moderate | Low | | Low | | High | | High | | Low | |  |
| Vertical center anterior angle on oblique view (VCA)[[18](#_ENREF_18)] | Progression was defined in 3 different definitions: 1. ≥1 K-L grade increase 2. ≥1 JSN score increase 3. Time to THP. No significant association was found between VCA and K-L grade or JSN score. HR 0.91 (95% CI 0.87; 0.95) was found for each degree increase in VCA and time to THP in univariate analysis, in multivariate analysis this association was not significant. | Low | High | | Moderate | | Moderate | | Low | | Low | |  |
| Acetabular depth angle (AD)[[18](#_ENREF_18)] | Progression was defined in 3 different definitions: 1. ≥1 K-L grade increase 2. ≥1 JSN score increase 3. Time to THP. No significant association was found between AD and K-L grade or JSN score. HR 0.99 (95% CI 0.87; 1.13) was found for each degree increase in AD and time to THP in univariate analysis. | Low | High | | Moderate | | Moderate | | Low | | Low | |  |
| Joint effusion on ultrasound[[10](#_ENREF_10)] | Progression was THR. OR was 1.3 (p=0.49) in univariate analysis. | Low | Low | | Moderate | | Low | | Low | | Low | |  |
| Fluid around tendon on ultrasound[[10](#_ENREF_10)] | Progression was THR. OR was smaller than 0.01 (p=0.68) in univariate analysis. | Low | Low | | Moderate | | Low | | Low | | Low | |  |
| Fluid around trochanteric bursa on ultrasound[[10](#_ENREF_10)] | Progression was THR. OR was 0.01 (p=0.76) in univariate analysis. | Low | Low | | Moderate | | Low | | Low | | Low | |  |

^ref^= reference of the study in which prognostic factor is described. JSN: joint space narrowing. OR: odds ratio. HR: hazard ratio. RR: relative risk. 95% CI: 95% confidence interval. THR: total hip replacement. LCGA: latent class growth analysis. YMN: yearly mean narrowing. JSW: joint space width. JSN: joint space narrowing. BMC: bone mineral content. Significant association means statistically significant at p<0.05.

References

1. Reijman M, Hazes JMW, Pols H, Bernsen RMD, Koes BW, Bierma-Zeinstra SMA: **Role of radiography in predicting progression of osteoarthritis of the hip: Prospective cohort study**. *Br Med J* 2005, **330**(7501):1183-1185.

2. Peters TJ, Sanders C, Dieppe P, Donovan J: **Factors associated with change in pain and disability over time: A community-based prospective observational study of hip and knee osteoarthritis**. *Br J Gen Pract* 2005, **55**(512):205-211.

3. Hawker GA, Guan J, Croxford R, Coyte PC, Glazier RH, Harvey BJ, Wright JG, Williams JI, Badley EM: **A prospective population-based study of the predictors of undergoing total joint arthroplasty**. *Arthritis Rheum* 2006, **54**(10):3212-3220.

4. Golightly YM, Allen KD, Helmick CG, Schwartz TA, Renner JB, Jordan JM: **Hazard of incident and progressive knee and hip radiographic osteoarthritis and chronic joint symptoms in individuals with and without limb length inequality**. *J Rheumatol* 2010, **37**(10):2133-2140.

5. Verkleij SPJ, Hoekstra T, Rozendaal RM, Waarsing JH, Koes BW, Luijsterburg PAJ, Bierma-Zeinstra SMA: **Defining discriminative pain trajectories in hip osteoarthritis over a 2-year time period**. *Ann Rheum Dis* 2012, **71**(9):1517-1523.

6. Conrozier T, Jousseaume CA, Mathieu P, Tron AM, Caton J, Bejui J, Vignon E: **Quantitative measurement of joint space narrowing progression in hip osteoarthritis: A longitudinal retrospective study of patients treated by total hip arthroplasty**. *Br J Rheumatol* 1998, **37**(9):961-968.

7. Hoeven TA, Kavousi M, Clockaerts S, Kerkhof HJM, Van Meurs JB, Franco O, Hofman A, Bindels P, Witteman J, Bierma-Zeinstra S: **Association of atherosclerosis with presence and progression of osteoarthritis: The Rotterdam Study**. *Ann Rheum Dis* 2013, **72**(5):646-651.

8. Ledingham J, Dawson S, Preston B, Milligan G, Doherty M: **Radiographic progression of hospital referred osteoarthritis of the hip**. *Ann Rheum Dis* 1993, **52**(4):263-267.

9. Kalyoncu U, Gossec L, Nguyen M, Berdah L, Mazieres B, Lequesne M, Dougados M: **Self-reported prevalence of psoriasis and evaluation of the impact on the natural history of hip osteoarthritis: Results of a 10 years follow-up study of 507 patients (ECHODIAH study)**. *Jt Bone Spine* 2009, **76**(4):389-393.

10. Lievense AM, Koes BW, Verhaar JAN, Bohnen AM, Bierma-Zeinstra SMA: **Prognosis of hip pain in general practice: A prospective followup study**. *Arthritis Care Res* 2007, **57**(8):1368-1374.

11. Dorleijn DMJ, Luijsterburg PAJ, Bay-Jensen AC, Siebuhr AS, Karsdal MA, Rozendaal RM, Bos PK, Bierma-Zeinstra SMA: **Association between biochemical cartilage markers and clinical symptoms in patients with hip osteoarthritis: Cohort study with 2-year follow-up**. *Osteoarthr Cartilage* 2015, **23**(1):57-62.

12. Lane NE, Nevitt MC, Lui LY, De Leon P, Corr M: **Wnt signaling antagonists are potential prognostic biomarkers for the progression of radiographic hip osteoarthritis in elderly Caucasian women**. *Arthritis Rheum* 2007, **56**(10):3319-3325.

13. Chevalier X, Conrozier T, Gehrmann M, Claudepierre P, Mathieu P, Unger S, Vignon E: **Tissue inhibitor of metalloprotease-1 (TIMP-1) serum level may predict progression of hip osteoarthritis**. *Osteoarthr Cartilage* 2001, **9**(4):300-307.

14. Nelson AE, Golightly YM, Kraus VB, Stabler T, Renner JB, Helmick CG, Jordan JM: **Serum transforming growth factor-beta 1 is not a robust biomarker of incident and progressive radiographic osteoarthritis at the hip and knee: The Johnston County Osteoarthritis Project**. *Osteoarthr Cartilage* 2010, **18**(6):825-829.

15. Conrozier T, Saxne T, Fan CSS, Mathieu P, Tron AM, Heinegard D, Vignon E: **Serum concentrations of cartilage oligomeric matrix protein and bone sialoprotein in hip osteoarthritis: A one year prospective study**. *Ann Rheum Dis* 1998, **57**(9):527-532.

16. Fukushima K, Inoue G, Uchida K, Fujimaki H, Miyagi M, Nagura N, Uchiyama K, Takahira N, Takaso M: **Relationship between synovial inflammatory cytokines and progression of osteoarthritis after hip arthroscopy: Experimental assessment**. *J Orthop Surg* 2018, **26**(2).

17. Birrell F, Afzal C, Nahit E, Lunt M, Macfarlane GJ, Cooper C, Croft PR, Hosie G, Silman AJ: **Predictors of hip joint replacement in new attenders in primary care with hip pain**. *Br J Gen Pract* 2003, **53**(486):26-30.

18. Bouyer B, Mazieres B, Guillemin F, Bouttier R, Fautrel B, Morvan J, Pouchot J, Rat AC, Roux CH, Verrouil E *et al*: **Association between hip morphology and prevalence, clinical severity and progression of hip osteoarthritis over 3 years: The knee and hip osteoarthritis long-term assessment cohort results**. *Jt Bone Spine* 2016, **83**(4):432-438.
