# Supplementary material for: Prognostic factors for progression of osteoarthritis of the hip: a systematic review
Source: Arthritis Res Ther. 2019 Aug 23;21:192. doi: 10.1186/s13075-019-1969-9 (PMC6708123; doi:10.1186/s13075-019-1969-9)
Supplement: Supplementary file 5 — Factors predicting total hip replacement, clinical or radiological progression combined. (DOCX 82 kb) [file 13075_2019_1969_MOESM5_ESM.docx]

Additional file 5. Factors predicting total hip replacement, clinical or radiological progression combined.

| Prognostic factor | Studies | Associations | Best-evidence synthesis |
| --- | --- | --- | --- |
| *Patient variables* | | |  |
| *No association* |  |  |  |
| Height | | | Moderate evidence for no association |
|  | 1 low risk of bias cohort[[1](#_ENREF_1)]  1 cohort[[2](#_ENREF_2)] | No  No |  |
| Weight | | | Moderate evidence for no association |
|  | 1 low risk of bias cohort[[1](#_ENREF_1)]  2 cohorts[[2-4](#_ENREF_2)] | No  No, no |  |
| Smoking | | | Moderate evidence for no association |
|  | 1 low risk of bias cohort[[5](#_ENREF_5)]  1 cohort[[2](#_ENREF_2)] | No  No |  |
| Heavy physical work | | | Moderate evidence for no association |
|  | 1 low risk of bias cohort[[5](#_ENREF_5)]  1 cohort[[6](#_ENREF_6)] | No  No |  |
| *Conflicting evidence* | |  |  |
| Family history of hip OA | | | Conflicting evidence |
|  | 2 cohorts[[7](#_ENREF_7), [8](#_ENREF_8)] | Positive, no |  |
| *Disease characteristics* | | |  |
| *No association* |  |  |  |
| Painful hip external rotation (passive or active) | | | Moderate evidence for no association |
|  | 1 low risk of bias cohort[[9](#_ENREF_9)]  1 cohort[[10](#_ENREF_10)] | No  No |  |
| Painful hip adduction (passive or active) | | | Moderate evidence for no association |
|  | 1 low risk of bias cohort[[9](#_ENREF_9)]  1 cohort[[10](#_ENREF_10)] | No  No |  |
| Painful hip abduction (passive or active) | | | Moderate evidence for no association |
|  | 1 low risk of bias cohort[[9](#_ENREF_9)]  1 cohort[[10](#_ENREF_10)] | No  No |  |
| Hypertension | | | Limited evidence for no association |
|  | 2 cohorts[[6](#_ENREF_6), [11](#_ENREF_11)] | No, no |  |
| *Conflicting evidence* |  |  |  |
| Use of walking devices | | | Conflicting evidence |
|  | 1 low risk of bias cohort[[12](#_ENREF_12)]  1 cohort[[13](#_ENREF_13)] | No  Yes |  |
| Concurrent knee OA | | | Conflicting evidence |
|  | 2 cohorts[[6](#_ENREF_6), [14](#_ENREF_14)] | Positive, no |  |
| *Chemical or imaging markers* | | |  |
| *No association* |  |  |  |
| Level of vitamin D in serum | | | Moderate evidence for no association |
|  | 1 low risk of bias cohort[[15](#_ENREF_15)]  1 cohort[[16](#_ENREF_16)] | No  No |  |
| *Conflicting evidence* |  |  |  |
| Lateral or superolateral migration of the femoral head | | | Conflicting evidence |
|  | 1 low risk of bias cohort[[17](#_ENREF_17)]  1 cohort[[3](#_ENREF_3), [4](#_ENREF_4)] | No  Positive |  |

References

1. Pisters MF, Veenhof C, van Dijk GM, Heymans MW, Twisk JWR, Dekker J: **The course of limitations in activities over 5 years in patients with knee and hip osteoarthritis with moderate functional limitations: Risk factors for future functional decline**. *Osteoarthr Cartilage* 2012, **20**(6):503-510.

2. Conrozier T, Jousseaume CA, Mathieu P, Tron AM, Caton J, Bejui J, Vignon E: **Quantitative measurement of joint space narrowing progression in hip osteoarthritis: A longitudinal retrospective study of patients treated by total hip arthroplasty**. *Br J Rheumatol* 1998, **37**(9):961-968.

3. Danielsson LG: **Incidence and prognosis of coxarthrosis**. *Acta Orthop Scand Suppl* 1964, **66**:SUPPL 66:61-114.

4. Danielsson LG: **Incidence and prognosis of coxarthrosis**. *Clin Orthop Relat Res* 1993(287):13-18.

5. Holla JFM, Steultjens MPM, Roorda LD, Heymans MW, Ten Wolde S, Dekker J: **Prognostic factors for the two-year course of activity limitations in early osteoarthritis of the hip and/or knee**. *Arthritis Care Res* 2010, **62**(10):1415-1425.

6. Reijman M, Hazes JMW, Pols H, Bernsen RMD, Koes BW, Bierma-Zeinstra SMA: **Role of radiography in predicting progression of osteoarthritis of the hip: Prospective cohort study**. *Br Med J* 2005, **330**(7501):1183-1185.

7. Pollard TCB, Batra RN, Judge A, Watkins B, McNally EG, Gill HS, Arden NK, Carr AJ: **Genetic predisposition to the presence and 5-year clinical progression of hip osteoarthritis**. *Osteoarthr Cartilage* 2012, **20**(5):368-375.

8. Tron AM, Conrozier T, Mathieu P, Vignon E: **[Rate of joint space pinching in coxarthrosis] Vitesse de pincement de l'interligne articulaire dans la coxarthrose**. *Rev Rhum Ed Fr* 1994, **61**(9 Pt 2):124S-130S.

9. Bastick AN, Verkleij SPJ, Damen J, Wesseling J, Hilberdink WKHA, Bindels PJE, Bierma-Zeinstra SMA: **Defining hip pain trajectories in early symptomatic hip osteoarthritis - 5 year results from a nationwide prospective cohort study (CHECK)**. *Osteoarthritis Cartilage* 2016, **24**(5):768-775.

10. Lievense AM, Koes BW, Verhaar JAN, Bohnen AM, Bierma-Zeinstra SMA: **Prognosis of hip pain in general practice: A prospective followup study**. *Arthritis Care Res* 2007, **57**(8):1368-1374.

11. Peters TJ, Sanders C, Dieppe P, Donovan J: **Factors associated with change in pain and disability over time: A community-based prospective observational study of hip and knee osteoarthritis**. *Br J Gen Pract* 2005, **55**(512):205-211.

12. van Dijk GM, Veenhof C, Spreeuwenberg P, Coene N, Burger BJ, van Schaardenburg D, van den Ende CH, Lankhorst GJ, Dekker J: **Prognosis of Limitations in Activities in Osteoarthritis of the Hip or Knee: A 3-Year Cohort Study**. *Arch Phys Med Rehabil* 2010, **91**(1):58-66.

13. Birrell F, Afzal C, Nahit E, Lunt M, Macfarlane GJ, Cooper C, Croft PR, Hosie G, Silman AJ: **Predictors of hip joint replacement in new attenders in primary care with hip pain**. *Br J Gen Pract* 2003, **53**(486):26-30.

14. Juhakoski R, Malmivaara A, Lakka TA, Tenhonen S, Hannila ML, Arokoski JP: **Determinants of pain and functioning in hip osteoarthritis - a two-year prospective study**. *Clin Rehabil* 2013, **27**(3):281-287.

15. Laslett LL, Quinn S, Burgess JR, Parameswaran V, Winzenberg TM, Jones G, Ding C: **Moderate vitamin D deficiency is associated with changes in knee and hip pain in older adults: a 5-year longitudinal study**. *Ann Rheum Dis* 2014, **73**(4):697-703.

16. Bergink AP, Zillikens MC, Van Leeuwen JPTM, Hofman A, Uitterlinden AG, van Meurs JBJ: **25-Hydroxyvitamin D and osteoarthritis: A meta-analysis including new data**. *Semin Arthritis Rheum* 2016, **45**(5):539-546.

17. Gossec L, Tubach F, Baron G, Ravaud P, Logeart I, Dougados M: **Predictive factors of total hip replacement due to primary osteoarthritis: A prospective 2 year study of 505 patients**. *Ann Rheum Dis* 2005, **64**(7):1028-1032.
